# The epigenetic factor BORIS (*CTCFL*) controls the androgen receptor regulatory network in ovarian cancer

**DOI:** 10.1038/s41389-019-0150-2

**Published:** 2019-08-12

**Authors:** Marisol Salgado-Albarrán, Rodrigo González-Barrios, Lissania Guerra-Calderas, Nicolás Alcaraz, Thalía Estefanía Sánchez-Correa, Clementina Castro-Hernández, Yesennia Sánchez-Pérez, Elena Aréchaga-Ocampo, Alejandro García-Carrancá, David Cantú de León, Luis A. Herrera, Jan Baumbach, Ernesto Soto-Reyes

**Affiliations:** 1 Natural Sciences Department, Universidad Autónoma Metropolitana-Cuajimalpa (UAM-C), Mexico City, 05300 Mexico; 2 Chair of Experimental Bioinformatics, TUM School of Life Sciences Weihenstephan, Technical University of Munich, Munich, Germany; 3 Cancer Biomedical Research Unit, Instituto Nacional de Cancerología (INCan), Mexico City, Mexico; 4 The Bioinformatics Centre Section for RNA and Computational Biology, Department of Biology, University of Copenhagen, Copenhagen, Denmark; 5 Instituto Nacional de Neurología y Neurocirugía “Manuel Velasco Suárez”, Mexico City, Mexico

**Keywords:** Ovarian cancer, Tumour biomarkers, Epigenetics

## Abstract

The identification of prognostic biomarkers is a priority for patients suffering from high-grade serous ovarian cancer (SOC), which accounts for >70% of ovarian cancer (OC) deaths. Meanwhile, borderline ovarian cancer (BOC) is a low malignancy tumor and usually patients undergo surgery with low probabilities of recurrence. However, SOC remains the most lethal neoplasm due to the lack of biomarkers for early diagnosis and prognosis. In this regard, BORIS (*CTCFL*), a CTCF paralog, is a promising cancer biomarker that is overexpressed and controls transcription in several cancer types, mainly in OC. Studies suggest that BORIS has an important function in OC by altering gene expression, but the effect and extent to which BORIS influences transcription in OC from a genome-wide perspective is unclear. Here, we sought to identify BORIS target genes in an OC cell line (OVCAR3) with potential biomarker use in OC tumor samples. To achieve this, we performed in vitro knockout and knockdown experiments of BORIS in OVCAR3 cell line followed by expression microarrays and bioinformatics network enrichment analysis to identify relevant BORIS target genes. In addition, ex vivo expression data analysis of 373 ovarian cancer patients were evaluated to identify the expression patterns of BORIS target genes. In vitro, we uncovered 130 differentially expressed genes and obtained the BORIS-associated regulatory network, in which the androgen receptor (AR) acts as a major transcription factor. Also, *FN1*, *FAM129A*, and *CD97* genes, which are related to chemoresistance and metastases in OC, were identified. In SOC patients, we observed that malignancy is associated with high levels of BORIS expression while BOC patients show lower levels. Our study suggests that BORIS acts as a main regulator, and has the potential to be used as a prognostic biomarker and to yield novel drug targets among the genes BORIS controls in SOC patients.

## Introduction

The most frequent epithelial ovarian cancer (OC) type is serous ovarian cancer (SOC), which accounts for about 90% of the OC cases. On the other hand, borderline ovarian cancer (BOC), which shows a more favorable outcome, accounts for an estimated 15–20% of all ovarian neoplasms. However, SOC is the most lethal gynecological neoplasm due to the lack of early diagnosis, prognosis, treatment, and response biomarkers^1^. Approximately, 70% of the cases are diagnosed in late stages, where the disease has already disseminated and the survival rate is low^1–3^. Thus, the search for new cancer biomarkers is one of the main goals in OC research.

In this regard, one protein that has gained interest by its potential use as a biomarker is the Brother Of the Regulator of Imprinted Sites (BORIS)^4–7^. BORIS is a transcriptional factor coded by the *CTCFL* gene, a paralog of *CTCF*. BORIS shows a very specific expression pattern; for example, it is highly expressed in testicular germ cells, and has very low levels in other somatic tissues^8,9^. Remarkably, BORIS expression is reactivated in several neoplasms, such as lung, breast, prostate, and OC;^5,10–12^ where it participates in different cellular processes, such as cell proliferation and apoptosis^13,14^. Given the above, BORIS was proposed by the NCI as one of the most promising cancer antigens^15^.

Furthermore, BORIS shows high expression levels in OC patients and is strongly associated with poor prognosis, which suggests that BORIS, or the molecular network it controls, have a role in OC progression^16,17^. To address this question, great efforts have been made to understand the regulatory function of BORIS in gene expression; for example, in OC cells, BORIS can act as a transcriptional activator of the *hTERT* gene, known for its important contribution in cell immortalization^18^. However, the function of BORIS in OC is still not completely understood. There are no genome-wide unbiased studies of BORIS activity to understand the effect and the extent to which this protein influences transcription and oncogenic processes in OC. Thus, the identification and understanding of the regulatory function of BORIS could help to predict novel drug targets or to use it as a prognostic biomarker in OC patients.

Hence, in this study we aimed to investigate the regulatory role of BORIS in OC from a genome-wide perspective using gene expression microarrays and bioinformatics analyses in OC cell lines and patients’ samples. In vitro, we identified that BORIS negatively regulates the androgen receptor (*AR*) gene, as well as fibronectin 1 (*FN1*), family with sequence similarity 129-member A (*FAM129A*) and CD97 antigen (*CD97*) genes, which are commonly deregulated in OC patients and associated with poor prognosis. In addition, we evaluated the expression patterns of *CTCFL* (BORIS) and their related targets genes on publicly available OC patient data sets, which were subsequently validated in an independent set of samples obtained from SOC, BOC patients, and non neoplastic fresh tissue.

## Results

### Characterization of BORIS expression in OC cell lines and BORIS-deficient cells

**Fig. 1:**
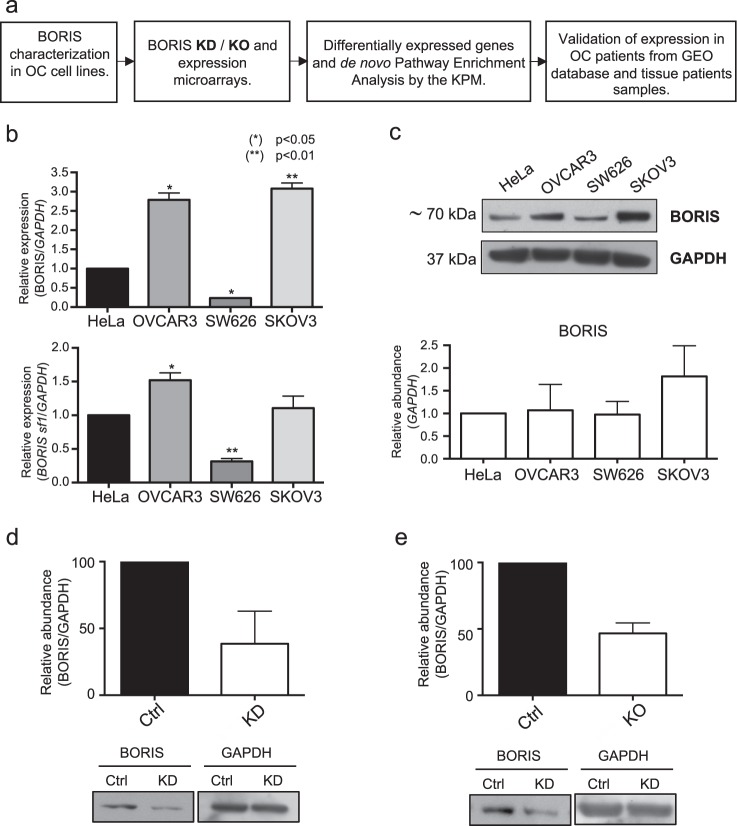
Characterization of BORIS expression in OC-derived cell lines. **a** Schematic workflow of the experimental and bioinformatics approaches employed. **b** Relative expression of BORIS mRNA transcripts and BORIS *sf1* subfamily of splicing variants. **c** Relative abundance of BORIS protein (~70 kDa) using HeLa cell line as a positive control (*n* = 3). **d** Western blot densitometry of BORIS KD efficiency after siRNA transfection versus negative Ctrl siRNA. Lower boxes show BORIS and GAPDH representative western blot bands. Three biological replicates were performed. **e** Densitometry of BORIS KO efficiency after CRISPR/Cas9 plasmid transient transfection versus non-targeting Ctrl CRISPR/Cas9. Lower panel shows BORIS and GAPDH representative western blot bands. Three biological replicates were performed. KD knockdown, KO knockout, Ctrl control

Given that *CTCFL* (BORIS) has been found to be mainly deregulated in OC samples compared with other types of neoplasms (Supplementary Fig. S1), the aim of our study was to explore the participation of BORIS in gene regulation in OC. This work was addressed by two different experimental approaches: in vitro using OC cell lines, molecular biology techniques, and bioinformatics tools; and an ex vivo approach to evaluate the clinical relevance of BORIS in samples from OC patients, publicly available in GEO database and in fresh tissue samples from OC (Fig. 1a).

As a first approach, we evaluated the transcript and protein levels of BORIS in a set of three OC-derived cell lines (OVCAR3, SKOV3, and SW626), to select the most suitable cell model to carry out the following experiments (Fig. 1b). The expression of BORIS had been previously reported in HeLa, therefore we used this cell line as a positive control^19^.

We evaluated all BORIS transcripts reported using oligonucleotides located in a region shared by all its splicing variants. Also, a second pair of primers was used to amplify only the BORIS subfamily 1 of splicing variants (*sf1*), because it encodes for the canonic protein^20^. We observed that OVCAR3 and SKOV3 cell lines have higher levels of BORIS expression compared with HeLa. In addition, the expression levels of *sf1* is higher in OVCAR3 cells than in HeLa and the other OC-derived cells (Fig. 1b). In addition, we evaluated by western blot the abundance of BORIS in the different cell lines compared to HeLa (Fig. 1c). We did not observe a significant difference in BORIS among cell lines.

Considering that we were interested in evaluating the effect of the absence of BORIS expression and its association with OC, we chose OVCAR3 for subsequent experiments.

To evaluate the expression profiles related to BORIS, we obtained two cellular models with decreased levels: one by siRNAs transfection (KD), and the second with the CRISPR/Cas9 system (KO). Then, we compared the endogenous protein levels (controls) with the KD and KO cells. The efficiencies of the decrease in KD cells were 40–90% (Fig. 1d), while the KO cells exhibit 50% decrease (Fig. 1e).

### Expression profile analysis in the KD and KO cells

**Fig. 2:**
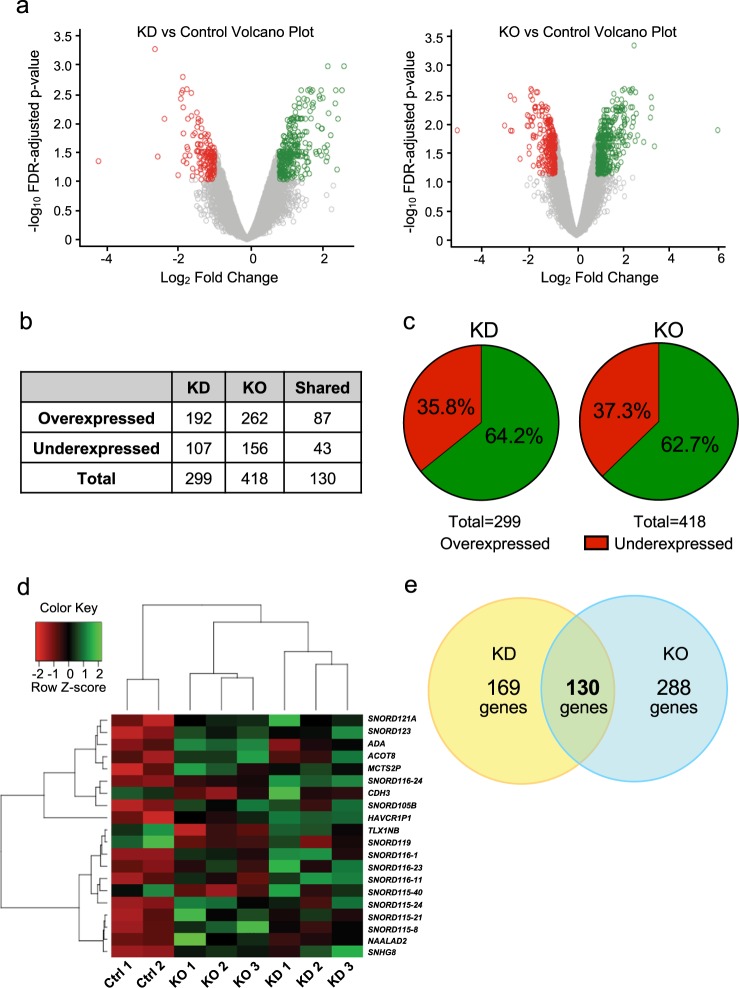
Differential expression microarray analysis of OVCAR3 controls, OVCAR3 BORIS KD, and OVCAR3 BORIS KO. **a** Volcano plots depicting differentially expressed genes in BORIS KD vs control cells and BORIS KO vs control cells. Selected differentially expressed genes are shown in colored circles (|fold change| ≥ 1.9 and FDR-adjusted *p*-value < 0.1). **b** The number of differentially expressed genes (overexpressed and underexpressed) identified in BORIS KD and BORIS KO cells. **c** Pie chart depicting the percentage of overexpressed and underexpressed genes in BORIS KD and KO cells. **d** Heatmap showing the top 20 genes that changed expression (Z-score) upon BORIS depletion (KD and KO). **e** Venn diagram showing the number of differentially expressed genes found in BORIS KD-only (yellow), BORIS KO-only (blue), and both BORIS KD and KO cells (green). KD knockdown, KO knockout

The first goal of this work was to identify novel genes regulated by BORIS in OC; thus, the expression profiles of the KD and KO cells versus controls were obtained with expression microarrays. Differentially expressed genes (DEG) in KD/controls and KO/controls were selected with FDR *p*-value < 0.1 and |fold change| > 1.9 (Fig. 2a). We observed that the number of DEG for the KD cells was 299 (192 overexpressed and 107 underexpressed; Supplementary Table 1) and 418 (262 overexpressed and 156 underexpressed; Supplementary Table 2) in the KO cells. GO-term enrichment analysis results was performed for both conditions (Supplementary Figs. S2, S3). In addition, we detected that 130 genes were consistent in both cellular models, where 87 genes were overexpressed and 43 genes underexpressed (Fig. 2b). In general, we identified that the decrease of BORIS in both cellular models (KD and KO) corresponds to an overexpression of 63% and a decrease of 36% of DEG, suggesting that BORIS may act mainly as a transcriptional repressor (Fig. 2c). Surprisingly, we observed that a group of noncoding genes (SNORDs) showed major expression changes after KD and KO (Fig. 2d).

With the DEG from the KD and KO cells, we select only the genes found in both experimental strategies. This was done to exclude those genes that could be a result of the experimental technique, and not because of BORIS decrease. We identified 130 DEG in both cellular models and the following studies were based on these genes (Fig. 2b, e).

### Identification of BORIS-associated network in the KD and KO cells

To identify a BORIS-associated regulatory network that provides us of potential novel BORIS targets in OC, we used the 130 DEG found in the KD and KO cells, and performed a *de novo* pathway enrichment analysis with KeyPathwayMiner (KPM)^21^. Given a biological network and set of expression studies, KPM extracts subnetworks enriched with differentially expressed genes (DEG). This method allowed us to discover previously uncharacterized regulatory networks by extracting a BORIS-associated network from HTRIdb^22^, a large experimentally validated human gene regulatory interaction database.

**Fig. 3:**
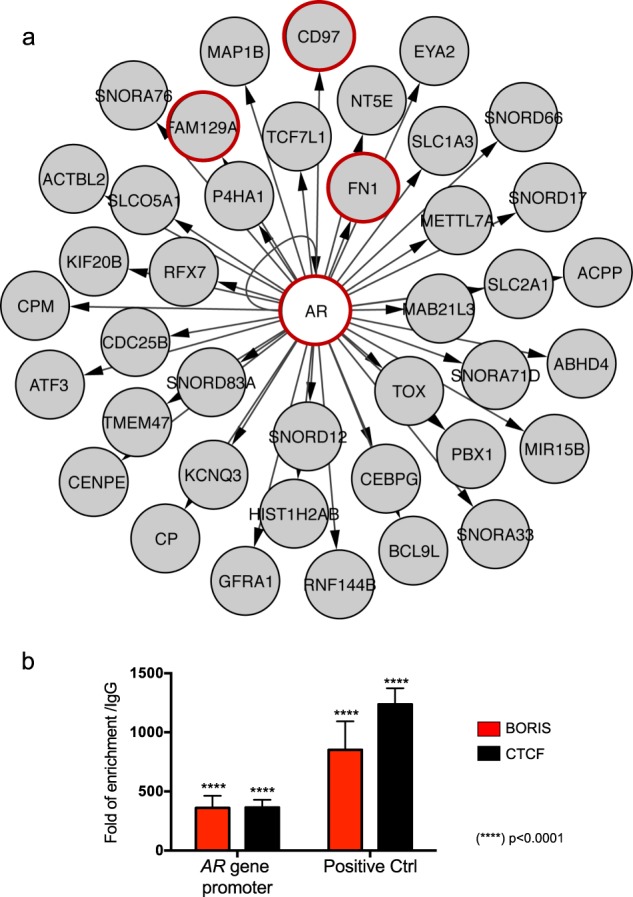
Identification and validation of *AR* as a target gene in the BORIS-associated transcriptional regulatory network. **a** BORIS-associated transcriptional regulatory network identified with KPM using differentially expressed genes found in BORIS KD and KO cells (139 genes). Gray circles indicate target genes, white circle shows the main transcription factor and arrows point the direction of transcriptional regulation. **b** qPCR evaluation of the products obtained from the chromatin immunoprecipitation assay of the *AR* promoter and positive control (*NY-ESO-1* promoter*)* precipitated with anti-BORIS antibody in OVCAR3 cells. As a positive control antibody, we used anti-CTCF, and as a negative control we used the IgG antibody

Our results show that the *AR* gene changes its expression upon BORIS depletion and is the main transcription factor that regulates the network (Fig. 3a). In addition, genes previously related to OC, such as *FN1*, *FAM129A*, and *CD97*, were identified as AR targets. Nevertheless, it was unclear whether BORIS regulates *AR* directly or indirectly. Thus, we performed a chromatin immunoprecipitation assay (ChIP) with an anti-BORIS antibody in the *AR* promoter. We observed that BORIS is located at the *AR* gene promoter (Fig. 3b).

**Fig. 4:**
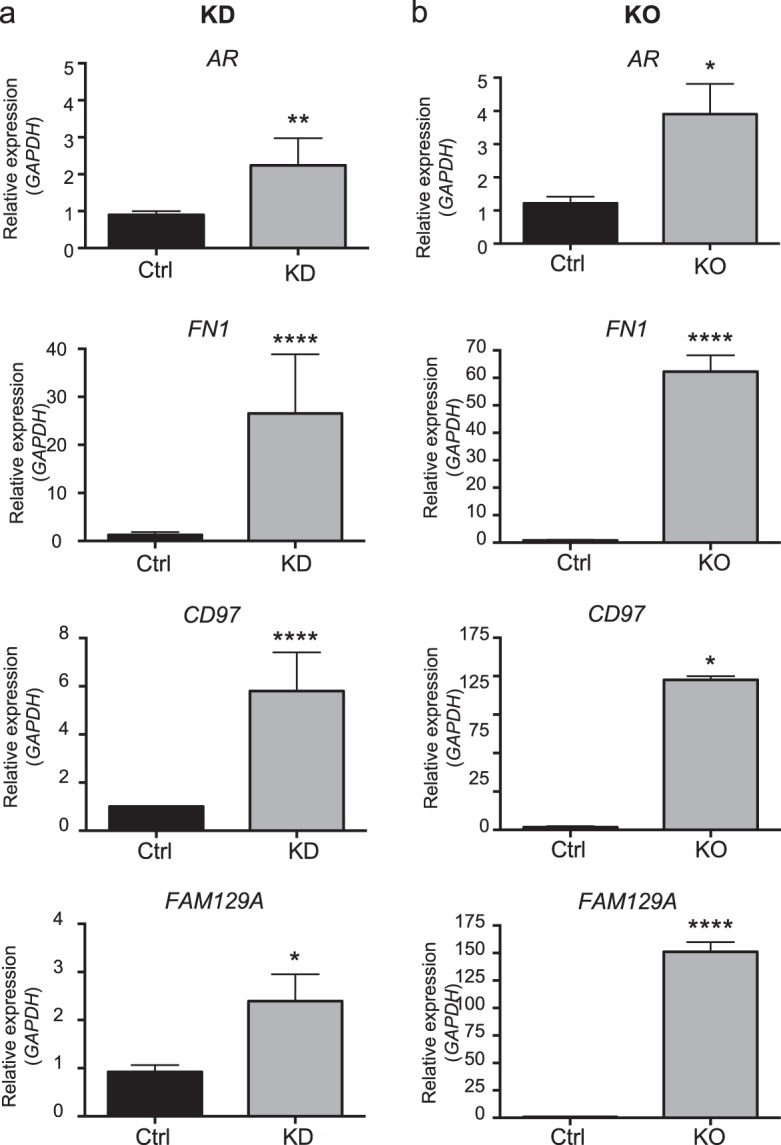
Validation of BORIS-associated transcriptional regulatory network. **a** RT-qPCR assays of *AR*, *FN1*, *CD97*, and *FAM129A* in KD and Ctrl cells. **b** RT-qPCR assays of *AR, FN1, CD97*, and *FAM129A* in KO and Ctrl cells. KD knockdown, KO knockout, Ctrl control

Finally, to validate the BORIS-associated network, we proceeded to evaluate the expression changes by qRT-PCR in the KD and KO cells. Our results show that, indeed, *AR*, *FN1*, *FAM129A*, and *CD97* genes change their expression levels after BORIS decrease (Figs. 4a, b). In accordance with our findings, expression data from The Cancer Cell Line Encyclopedia show that ~82% of the OC-derived cell lines reproduce the same phenomenon for *FN1*, *FAM129A*, and *CD97* genes (Supplementary Fig. S4). The latter proposes that BORIS acts as a transcriptional repressor of *AR*, *FN1*, *FAM129A*, and *CD97* genes, not only in OVCAR3 but also in a larger set of OC-derived cell lines, suggesting that this phenomenon might be related to OC biology.

### Expression analysis of *CTCFL* (BORIS), *AR, FN1*, and *FAM129A* and *CD97* genes in SOC, BOC, and non neoplastic samples

Previously, we identified that high levels of *AR*, *FN1*, *FAM129A*, and *CD97* transcripts are associated to a decrease in BORIS expression in OC cell lines. Thus, to determine if this expression pattern is similar in OC samples, we analyzed 343 expression microarrays from the GEO database (43 non neoplastic, 288 SOC, and 12 BOC samples).

**Fig. 5:**
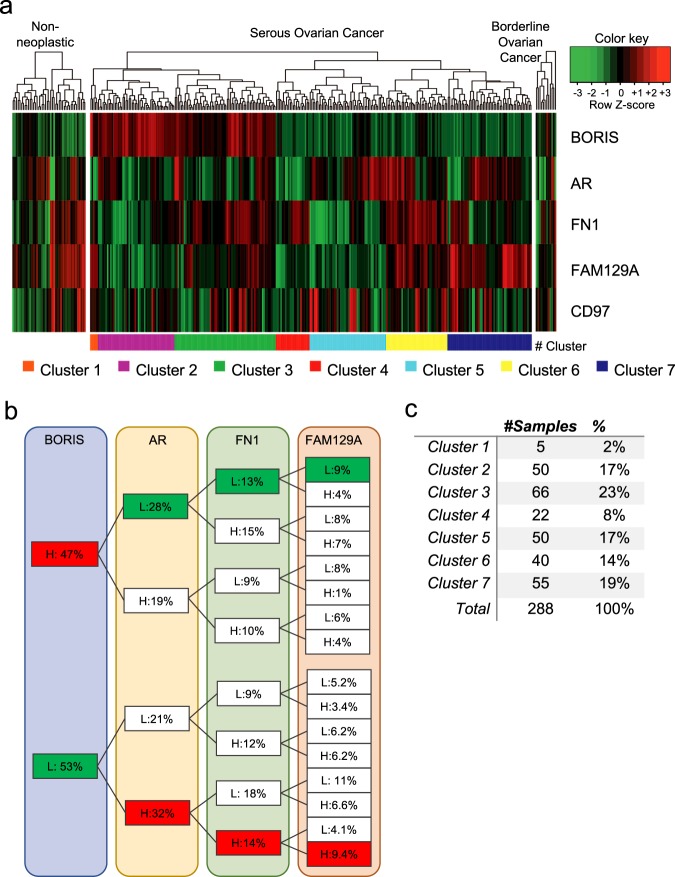
Expression profiles of *BORIS, AR, FN1, FAM129A*, and *CD97* genes in OC samples selected from the GEO database. **a** Hierarchical clustering of 343 samples (43 non neoplastic, 288 SOC, and 12 BOC samples) by BORIS, *AR*, *FN1*, *FAM129A*, and *CD97* expression levels (row Z-score). Columns show patient samples, and rows show genes. SOC samples are classified in seven clusters, shown in lower panel. **b** Percentage of the SOC samples with low or high BORIS, *AR*, *FN1*, and *FAM129A* expression. Percentage of samples with low BORIS and high *AR*, *FN1*, and *FAM129A* expression are highlighted in color, as well as the samples with high BORIS and low *AR*, *FN1*, and *FAM129A* expression. **c** Table of SOC sample frequencies classified on each cluster. OC ovarian cancer, H high, L low, SOC serous ovarian cancer, BOC borderline ovarian cancer

We extracted the expression values of *BORIS*, *AR*, *FN1*, *FAM129A*, and *CD97* genes and plotted heatmaps for non neoplastic, SOC, and BOC samples (Fig. 5a). Our data reveals a wide heterogeneity among normal and cancer samples. First, we observed two main dendrogram branches in SOC samples: BORIS positive (red) and BORIS negative (green); in contrast to non neoplastic and BOC samples, which show low levels of BORIS. An important subset of SOC patients exhibits high levels of BORIS (42–47%; Fig. 5a). Furthermore, in this group of patients, we can also find different expression combinations with *AR*, *FN1*, *FAM129A*, and *CD91*; however, an important group of samples with high levels of BORIS also show low levels of *AR* (28% of samples) along with low levels of *FN1* (13%; Fig. 5b), in agreement with our in vitro findings, suggesting that both genes might be playing an important role in SOC.

Furthermore, due to de incidence of SOC patients, we aimed to classify this type of OC into clusters based on the expression patterns of these genes (Fig. 5a, c). We identified a clear expression pattern in clusters number 2 and 6, which correspond to the phenomenon previously characterized in vitro in OVCAR3 cell line (Fig. 3), where the presence of BORIS is associated with low levels of *AR*, *FN1*, *FAM129A*, and *CD97* (Cluster 2), while the absence of BORIS has the opposite behavior (Cluster 6; Supplementary Figs. S5, S6).

In addition, we evaluated BORIS, *AR*, *FN1*, *CD97*, and *FAM129A* in tissue from patients’ samples to assess their clinical value. For instance, we wondered if the expression of these genes could be associated with a clinical feature. Thus, we obtained the relative expression levels of these genes in 11 SOC, 10 BOC, and 8 non neoplastic patients of a second cohort. We tested the results against clinical variables, and we identified that BORIS and *AR* genes are not associated with CA-125 expression, tumor size or FIGO stage (Table 1). However, we found that BORIS was associated with malignancy; for instance, SOC patients show significant higher levels of BORIS compared with BOC and non neoplastic samples (*p* = 0.017), in agreement with the previous finding in the large cohort from GEO database (Table 1). The later proposes BORIS as a relevant deregulated gene in OC, particularly in SOC, where a subset of patients show an inverse association between BORIS and AR expression, and is associated with tumor malignancy.

**Table 1:** Clinical and demographic characteristics of patients

			Expression				
*N* =	29		BORIS	AR	FN1	FAM129A	CD97
SOC	11	37.9%	***p*** **=** **0.017**	*p* = 1.000	*p* = 0.895	*p* = 0.195	*p* = 0.180
BOC	10	34.4%					
Non neoplastic	8	27.5%					
Age	*X* =	48.9 (21–81)	*p* = 0.793	*p* = 0.474	***p*** **=** **0.013**	***p*** **=** **0.013**	*p* = 0.483
	< 45 years	9 (31%)					
	> 45 years	20 (69%)					
Patient follow-up	38.3 months (1–80)						
Premenopause		12 (41%)	*p* = 0.428	*p* = 0.774	*p* = 0.876	*p* = 0.876	*p* = 0.432
Postmenopause		17 (59%)					
Number of births		2.3 (0–11)	*p* = 0.913	*p* = 0.507	***p*** **=** **0.017**	***p*** **=** **0.017**	*p* = 0.311
BMI	*X* =	26.7	*p* = 0.888	*p* = 0.663	***p*** **=** **0.018**	***p*** **=** **0.018**	*p* = 0.947
	SOC	27.3					
	BOC	26.5					
	Non neoplastic	26.2					
CA-125 Pre-Qx	*X* =	3649.1	*p* = 0.467	*p* = 0.488	*p* = 0.483	*p* = 0.586	*p* = 1.000
	SOC	478.2					
	BOC	9968.2					
	Non neoplastic	110.3					
Tumor size	*X* =	16.2	*p* = 0.208	*p* = 0.628	*p* = 0.812	*p* = 0.558	*p* = 0.466
	> 10 CM SOC	11 (100%)					
	> 10 CM BOC	7 (70%)					
Ascites	Yes	6 (28%)	*p* = 0.172	*p* = 0.565	*p* = 0.291	*p* = 0.457	*p* = 0.375
	No	15 (72%)					
Peritoneal implants	SOC	6 (54%)	*p* = 0.432	*p* = 0.183	*p* = 0.129	*p* = 0.382	*p* = 0.192
	BOC	2 (20%)					
FIGO	SOC	BOC					
I	0	5 (50%)	*p* = 0.510	*p* = 1.000	*p* = 0.523	*p* = 0.171	*p* = 0.142
II	3 (27%)	1 (10%)					
III	8 (73%)	4 (40%)					
IV	0	0					
Optimal cytoreduction	SOC	6 (54%)	*p* = 0.329	*p* = 0.236	*p* = 0.186	*p* = 0.112	*p* = 0.498
	BOC	100%					
Mortality	SOC	27%	*p* = 0.060	*p* = 0.420	*p* = 0.527	*p* = 0.636	*p* = 0.220
	BOC	0%					
	Non neoplastic	0%					

*p* < 0.05 is statistically significant

**Fig. 6:**
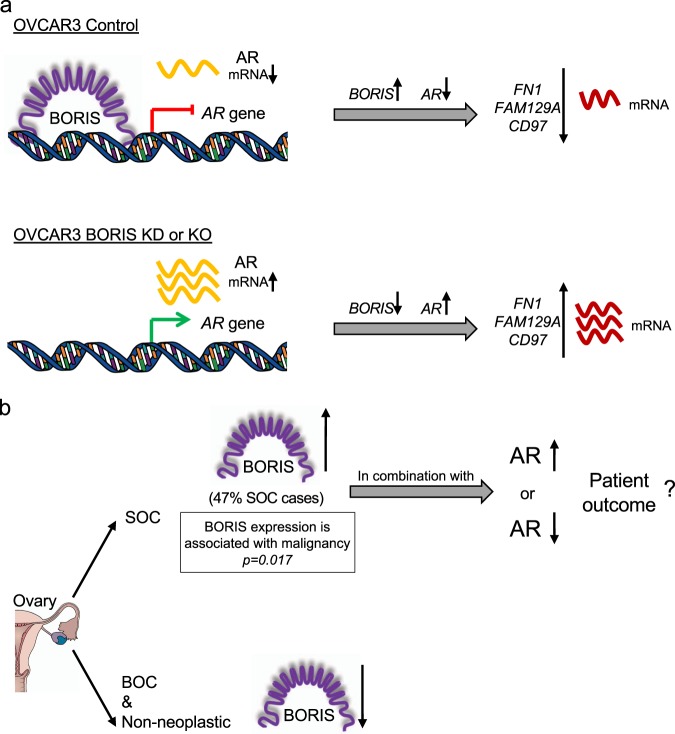
Schematic model of AR transcriptional repression mediated by BORIS and its clinical relevance in ovarian cancer patients. **a** BORIS is recruited to the *AR* gene promoter and mediates its transcriptional repression in OVCAR3 cell line. The AR levels reduction and the endogenous presence of BORIS have an effect in the *FN1*, *CD97*, and *FAM129A* gene repression. In contrast, in BORIS KO and KD cells, the absence of BORIS allows the *AR* gene activation, which could increase the transcript levels of *FN1*, *CD97*, and *FAM129A*. **b** A subset of SOC patients show high levels of BORIS, which is associated with malignancy in contrast to BOC and non neoplastic patients which have low levels of BORIS. OC ovarian cancer, BOC borderline ovarian cancer, SOC serous ovarian cancer, KO knockout, KD knockdown

In summary, our results show that BORIS binds to *AR* gene promoter and acts as a transcriptional repressor of *AR*. In addition, the decrease of BORIS levels is associated with an increase in *FN1*, *FAM129A*, and *CD97* gene expression (Fig. 6a). Furthermore, in OC patients, BORIS is overexpressed in 47% of SOC samples, and is downregulated in borderline and non neoplastic samples, thus it is significantly associated with malignancy. Our data also indicate that a fraction of SOC samples with overexpression of BORIS (47%) can show either underexpression (28%) or overexpression (19%) of *AR*, indicating that in OC samples, the relationship between BORIS and AR found in OVCAR3 cell line might not necessarily happen in all OC samples (Fig. 6b). Nevertheless, we still ignore whether the different combinations of BORIS and AR expression levels are associated with a specific outcome, and if their joint use as prognostic biomarkers is beneficial for SOC patients. In this regard, further studies are needed to address if BORIS and AR can be used together as prognostic biomarkers in OC.

## Discussion

SOC is as one of the most lethal gynecologic malignancies worldwide, and we lack effective biomarkers to improve the outcome^23^. Currently, only CA-125 and HE4 antigens are used in the medical practice, but they lack sensitivity^24^. Thus, the identification of novel tumor biomarkers that could benefit patients’ outcome is a priority in OC research. One of the proteins that has gained interest, given its potential use as a cancer biomarker, is BORIS^5,6^. This protein was proposed as a priority biomarker by the National Cancer Institute, because its deregulation is related to oncogenic properties, and as a biomarker could be used for its specificity and immunogenicity^15^. Previously, some studies have shown that the decrease of BORIS is associated with decreasing cell proliferation and cell viability^6,25^. Hence, we aimed to identify genes regulated by BORIS, which could provide some insights in the biology of OC. An analysis of differential expression exhibits that several small nuclear and small nucleolar RNAs are the most affected genes in the absence of BORIS. To understand the function of BORIS from a systems perspective, we applied KPM to integrate our expression studies with known gene regulatory interactions to extract novel disease pathways and potential drug targets^26^. Enriched with the 130 DEG, we identified and experimentally validated the *AR* gene as one of the main regulators of the BORIS-associated regulatory network. This type of analysis may suggest that BORIS could have its effect on transcriptional regulation by altering the levels of AR through its binding to the AR promoter. Our data show biological and therapeutic relevance, since AR has been used as a biomarker of different neoplasms, such as prostate cancer, and is an important druggable target^27^. Because the ovary is also responsible for the synthesis of AR, it would be of great importance the search for biomarkers related to this protein^28–30^. Interestingly, our results from KPM demonstrated that BORIS is partly responsible for the regulation of *AR* gene expression, and in turn, *AR* establishes a gene regulatory network with genes such as *FN1*, *CD97*, and *FAM129A*, which are related with poor prognosis, chemoresistance, and metastasis in several cancers, including OC^31–33^. Previous studies are limited to the identification of possible target genes by evaluating changes in gene expression by increasing or decreasing BORIS levels;^5,6,11,19,34^ however, our *de novo* pathway enrichment analysis allowed us to perform a novel selection (based on hundreds of experimental data available in public databases) of potential genes relevant in cancer, which are closely related through AR and BORIS. In conclusion, we found that in both, BORIS KD and KO models, BORIS regulates several genes, functioning mainly as a transcriptional repressor. Remarkably, BORIS acts as a transcriptional repressor of *AR* gene and binds to its promoter. The latter is of great relevance given the importance of *AR* deregulation in the development and malignancy of many types of cancer, also due to the current use of *AR* gene as a prognostic biomarker and therapy target in cancer, such as prostate cancer. In addition, our findings show that BORIS can also regulate other genes involved in OC, such as *FN1, FAM129A*, and *CD97*. Finally, we found a subset of SOC patients that show BORIS overexpression, which is significantly associated with malignancy.

Collectively, our in vitro and ex vivo studies confirm that BORIS has the potential to be used as a prognostic biomarker in SOC and to yield novel druggable targets among the regulatory network that BORIS controls.

## Materials and methods

### Cell lines culture

The cancer cell lines OVCAR3, SKOV3, SW626, and HeLa were obtained from ATCC (Manassas, VA, USA), and subcultured as described by the supplier.

### Knockdown and Knockout assays

To knockdown (KD) expression of BORIS, OVCAR3 cells were transfected with a pool of small interfering RNAs (siRNAs) (Cat. A-003819-100-0005, Dharmacon, Lafayette, CO, USA) or non-targeting control siRNA (Cat. D-001910-10-10, Dharmacon) and were incubated for 48 h in Accell Delivery Media (Cat. B-005000, Dharmacon). The assays were performed by triplicate.

Knockout (KO) of BORIS expression was performed by transfection of OVCAR3 cells with 2 μg of BORIS (Cat. sc-403313, SCBT, Dallas, TX, USA) or control CRISPR/Cas9 (Cat. Sc-418922, SCBT) plasmids. The assays were performed by triplicate. Following experiments were performed 24 h post transfection.

### Western blot assays

Western blot assays were performed with 30 μg of total protein. Primary and secondary antibodies used are described in Supplementary Table 3. Canonic BORIS abundance was determined by standard densitometry analysis, using ImageJ software (NIH, USA) with GAPDH as normalizing protein.

### RNA extraction, cDNA, and qRT-PCR analysis

The total RNA was extracted with TRIzol Reagent (Cat. 15596026, Invitrogen, Carlsbad, CA, United States), and the integrity and quality were analyzed with TapeStation 2200. cDNA was obtained with the GeneAmp PCR Core Kit (Cat. N8080143, Thermo Scientific, Waltham, MA, USA) using oligo(dT). qRT-PCR assays were performed on StepOnePlus using SYBR Green Master Mix (Cat. 4309155, Applied Biosystems, Foster City, CA, USA). All data were normalized to *GAPDH* using the ^ΔΔ^C_t_ method. Primers used are described in Supplementary Table 4. Experiments were performed by triplicate.

### Microarray analysis: hybridization and analysis

The GeneChip Human Gene 2.0 ST oligonucleotide arrays (Affymetrix, Santa Clara, CA, USA) were hybridized with two control samples of OVCAR3, three samples of OVCAR3 KD, and three samples of OVCAR3 KO cells, according to the instructions provided by the manufacturer.

Data normalization was performed with robust multiarray analysis (RMA). DEG were selected with “Limma” package^35^ and those with |fold change| > 1.9 and FDR-adjusted *p*-value < 0.1 were selected for further downstream analysis. *De novo* pathway enrichment analysis was performed with KPM^21^ with the Greedy search algorithm and the INES search strategy. We used as input of KPM, a regulatory network constructed from 51871 interactions found in the human transcriptional regulation interaction database (HTRIdb)^22^. Microarrays data have been deposited at the National Center for Biotechnology Information Gene Expression Omnibus (NCBI GEO Series Accession # GSE130163).

### Chromatin immunoprecipitation assays (ChIP)

OVCAR3 cells were cultured at 80% of confluence, chromatin was extracted according to the OneDay ChIP Kit (Cat. kch-oneDIP- 180, Diagenode, Denville, NJ, USA). Two independent chromatin preparations were analyzed. As a negative control, we used an IgG antibody provided by the kit. The antibodies and primers used are listed in Supplementary Tables 3 and 4.

### Patients samples

The ovarian tumor samples were collected from patients undergoing surgical resections at the INCan (Mexico City, Mexico) with previous written consent and the approval of the ethical committee (approval number 015/037/ICI). We analyzed a total of 21 samples from patients diagnosed with OC (11 SOC, 10 BOC), and eight non neoplastic ovarian samples obtained from January 1st, 2014 to December 31st, 2016. Demographics, prognostic markers, and epidemiologic exposure variables were obtained from the medical record.

### RNA extraction from patient samples

The tissue from patients was stored in RNAlater RNA Stabilization Reagent (Cat. AM7020, Invitrogen) at −20 °C. RNA from samples was obtained according to Peña-Llopis et al.^36^.

### GEO expression microarray analysis

Gene expression data from OC patients, evaluated by GeneChip Human Genome U133A 2.0 Plus arrays, were obtained from the GEO (Accession IDs: GSE14001, GSE18520, GSE19352, GSE36668, GSE38666, GSE63885, GSE40595 and GSE26193)^37–45^. A total of 343 samples were analyzed (43 from non neoplastic samples, 288 were from SOC samples, and 12 BOC patients). Batch effects were corrected with ComBat function from the “sva” package^46^. Then, normalized expression values were obtained for BORIS, *AR*, *FN1*, *FAM129A*, and *CD97* genes. Heatmaps were generated using the “ComplexHeatmap” package^47^, clustering was performed with hierarchical clustering where the optimal number of clusters was selected using the “NbClust” package^48^.

### Statistical analysis

The data from cell lines are shown as the mean ± standard error. The differences between groups were analyzed with a paired Student’s *t* test comparing the samples with the controls. Differences between groups were considered statistically significant when *p* < 0.05, *p* < 0.01, *p* < 0.001, and *p* < 0.0001.

For patient samples, we performed Chi-square test and Fisher’s exact test using STATA software version 13.0 software (StataCorp, TX, USA), to assess the relationship between BORIS, AR, FN1, FAM129A, and CD97 with clinicopathological characteristics. *P* < 0.05 was considered statistically significant.

## Supplementary information

Supplementary Figure S1

Supplementary Figure S2

Supplementary Figure S3

Supplementary Figure S4

Supplementary Figure S5

Supplementary Figure S6

Supplementary Table 1

Supplementary Table 2

Supplementary Table 3

Supplementary Table 4
