# Supplementary figures and images for: The epigenetic factor BORIS (CTCFL) controls the androgen receptor regulatory network in ovarian cancer
Source: Oncogenesis. 2019 Aug 12;8(8):41. doi: 10.1038/s41389-019-0150-2 (PMC6690894; doi:10.1038/s41389-019-0150-2)

RNA expression overview (TCGA dataset)

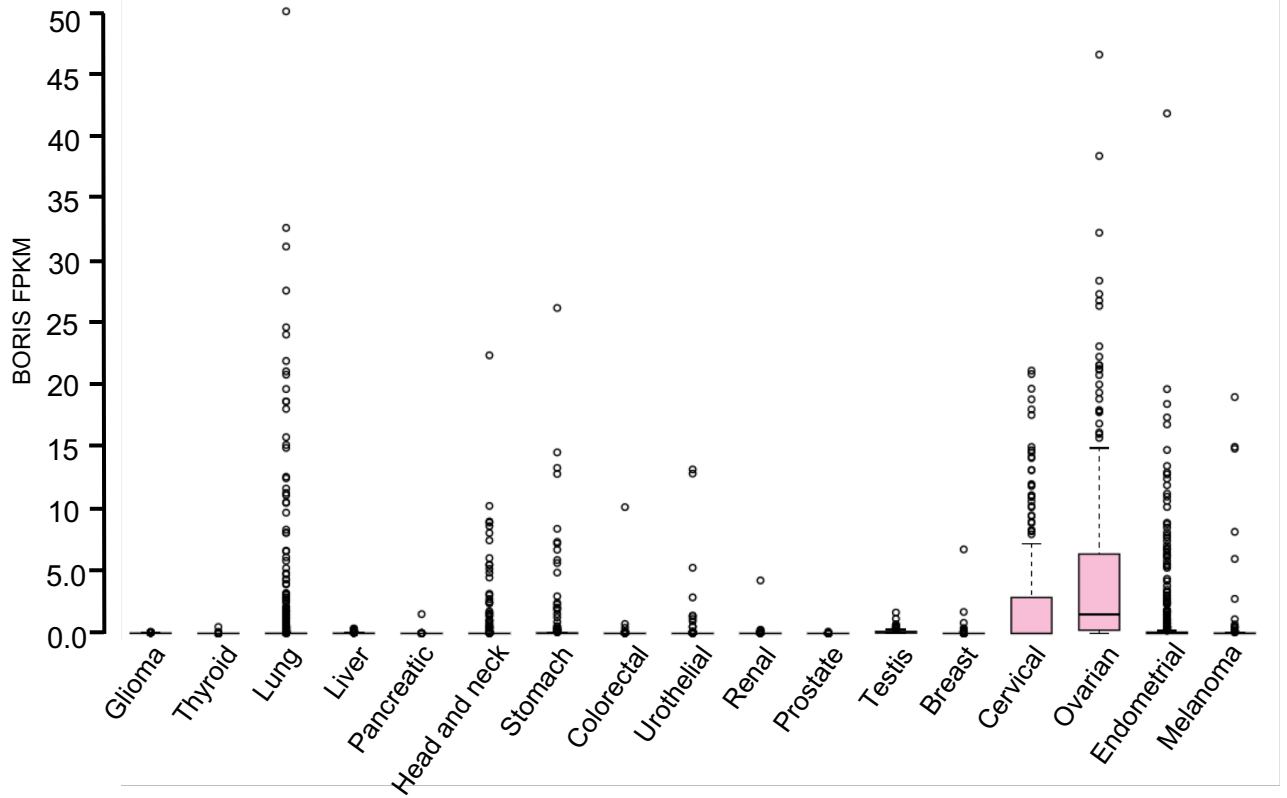

Figure S1

Supplement: Supplementary file 1 — Supplementary Figure S1 [file 41389_2019_150_MOESM1_ESM.pdf]

# BORIS Knockdown vs Control

## GO term Enrichment analysis

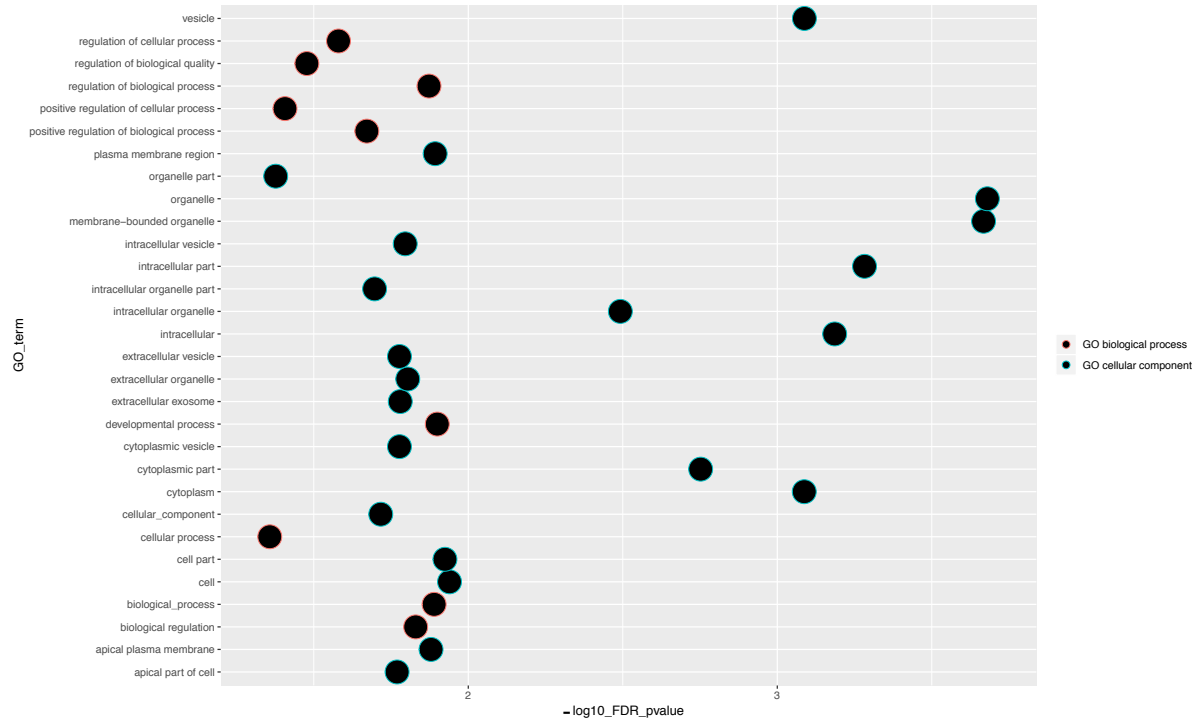

Figure S2

Supplement: Supplementary file 2 — Supplementary Figure S2 [file 41389_2019_150_MOESM2_ESM.pdf]

BORIS Knockout vs Control

GO term Enrichment analysis

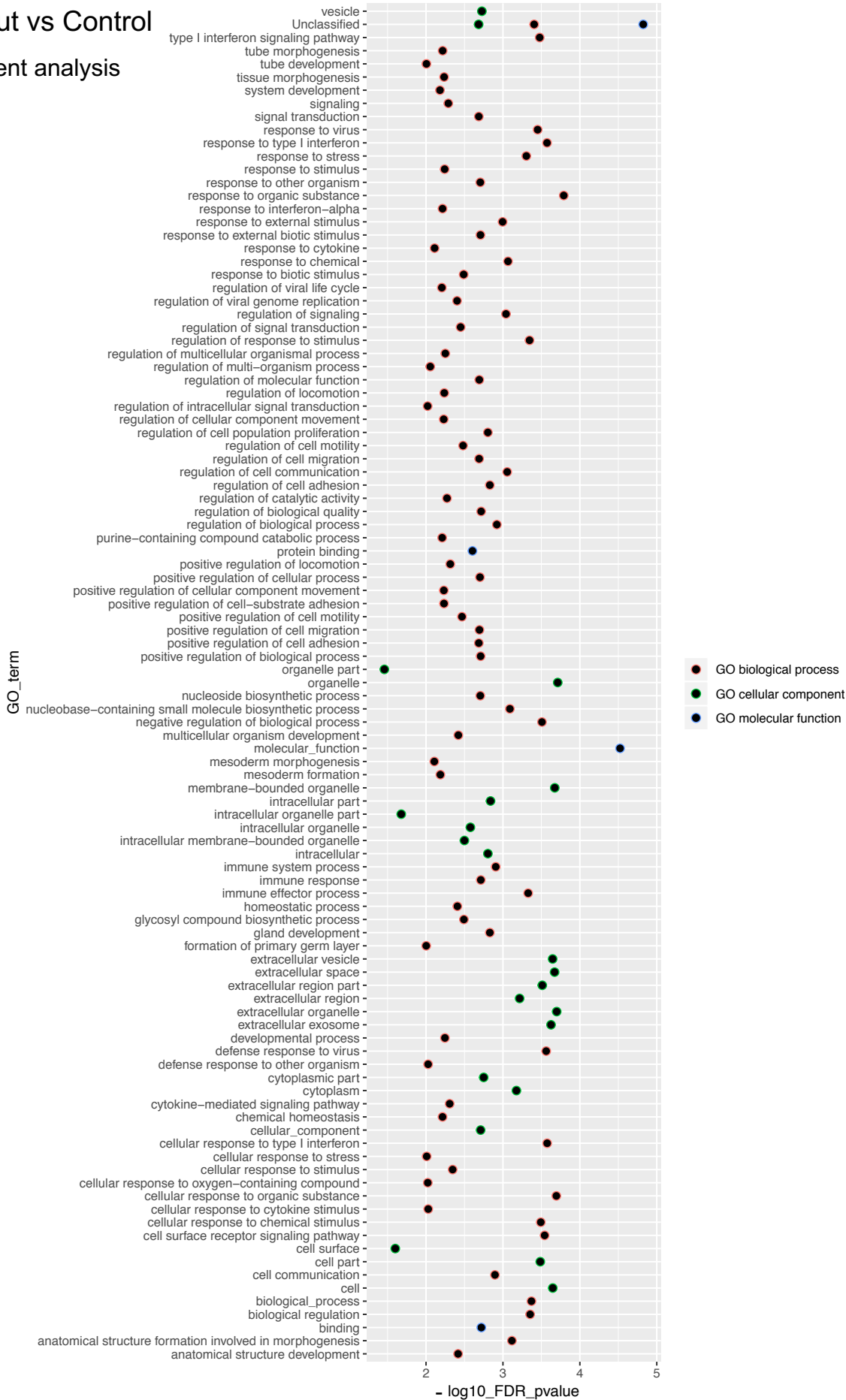

Figure S3

Supplement: Supplementary file 3 — Supplementary Figure S3 [file 41389_2019_150_MOESM3_ESM.pdf]

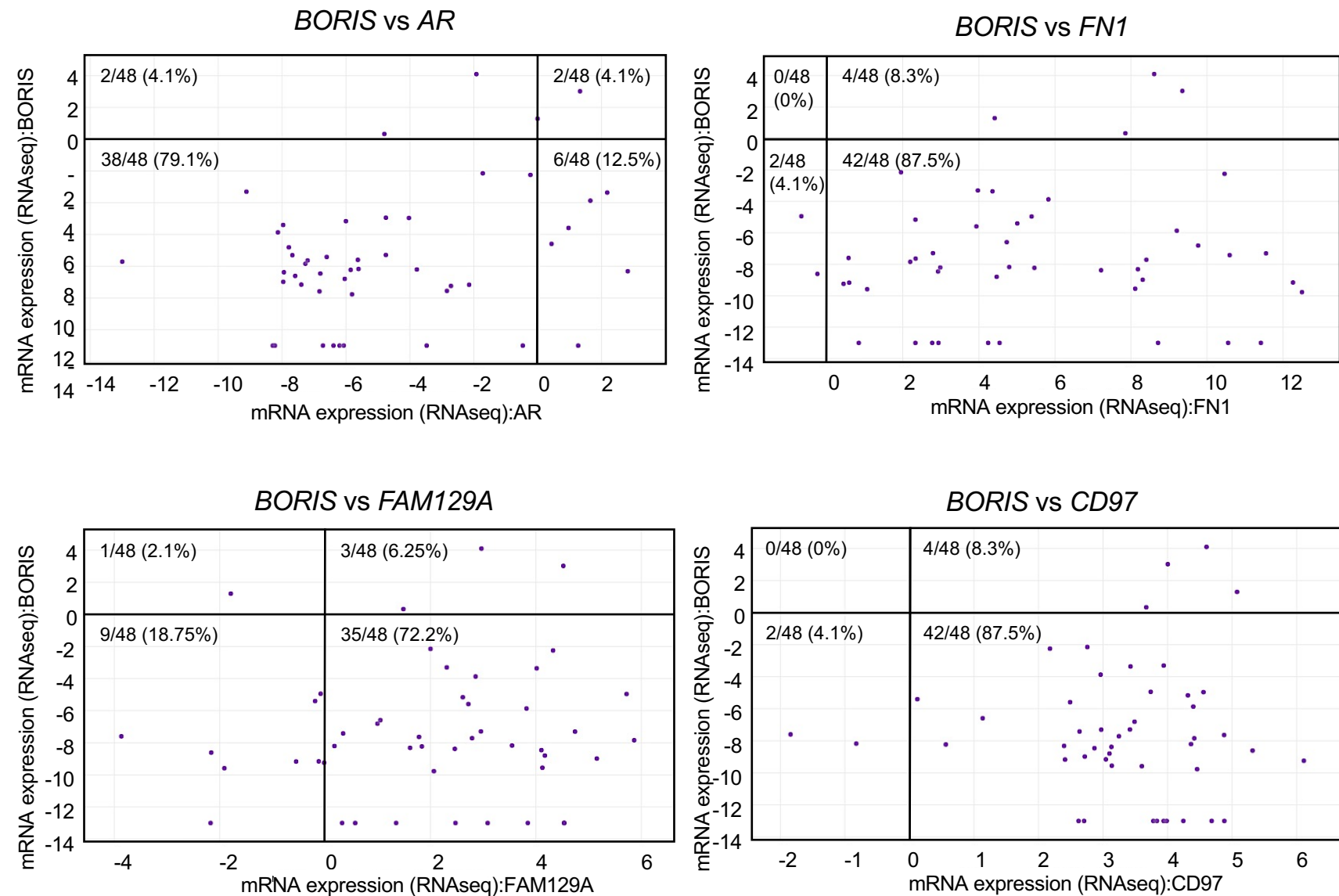

Figure S4

Supplement: Supplementary file 4 — Supplementary Figure S4 [file 41389_2019_150_MOESM4_ESM.pdf]

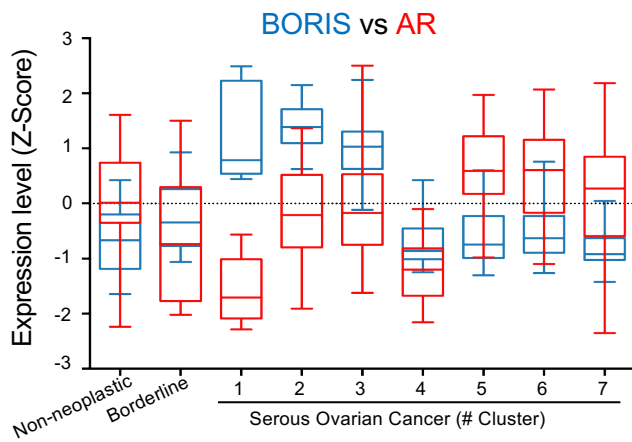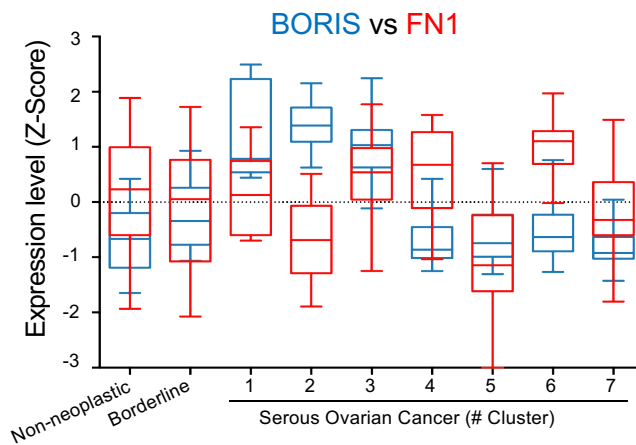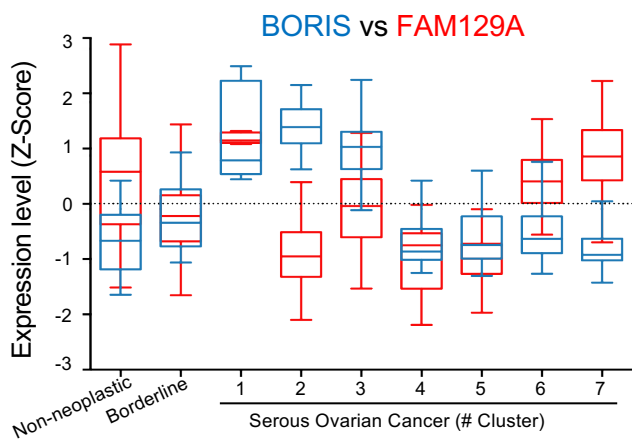

Figure S5

Supplement: Supplementary file 5 — Supplementary Figure S5 [file 41389_2019_150_MOESM5_ESM.pdf]

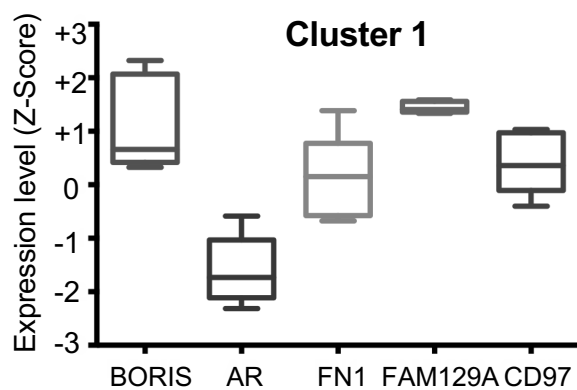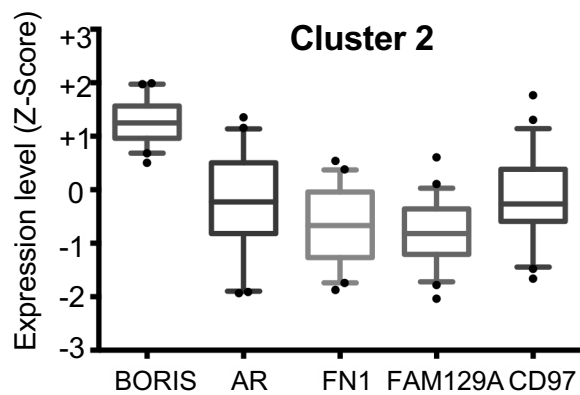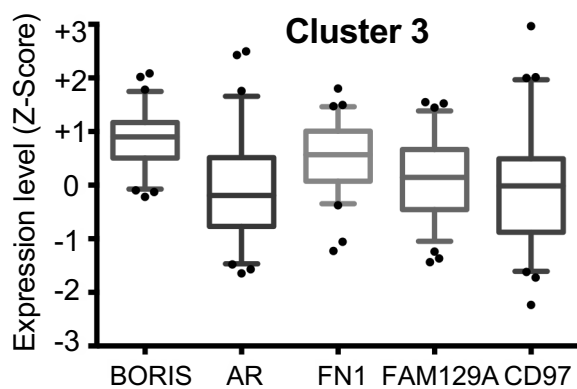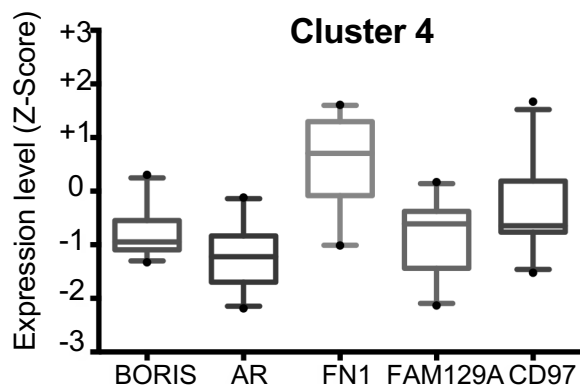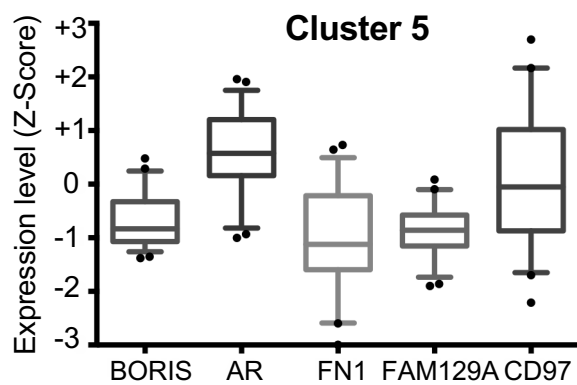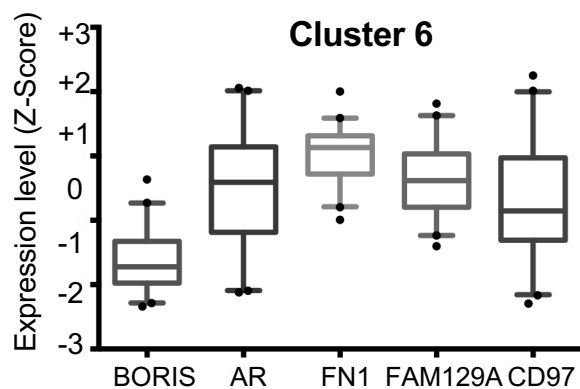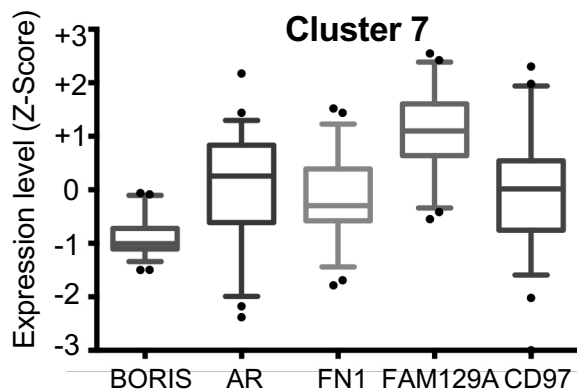

Figure S6

Supplement: Supplementary file 6 — Supplementary Figure S6 [file 41389_2019_150_MOESM6_ESM.pdf]
