# Supplementary material for: The epigenetic factor BORIS (CTCFL) controls the androgen receptor regulatory network in ovarian cancer
Source: Oncogenesis. 2019 Aug 12;8(8):41. doi: 10.1038/s41389-019-0150-2 (PMC6690894; doi:10.1038/s41389-019-0150-2)
Supplement: Supplementary file 7 — Supplementary Table 1 [file 41389_2019_150_MOESM7_ESM.docx]

Supplementary Table 1. Differentially expressed genes identified in BORIS Knockdown cells versus Control.

| ENTREZID | SYMBOL | GENENAME | log2FC | adj.P.Val |
| --- | --- | --- | --- | --- |
| 116496 | FAM129A | family with sequence similarity 129 member A | -2.462129519 | 0.00084076 |
| 3786 | KCNQ3 | potassium voltage-gated channel subfamily Q member 3 | -2.019685687 | 0.01225316 |
| 167410 | LIX1 | limb and CNS expressed 1 | -1.831916387 | 0.00308967 |
| 57631 | LRCH2 | leucine-rich repeats and calponin homology (CH) domain containing 2 | -1.809764373 | 0.0482869 |
| 83604 | TMEM47 | transmembrane protein 47 | -1.774633887 | 0.00133784 |
| 9076 | CLDN1 | claudin 1 | -1.729481143 | 0.05579107 |
| 25878 | MXRA5 | matrix-remodelling associated 5 | -1.667564272 | 0.01889415 |
| 4131 | MAP1B | microtubule associated protein 1B | -1.666375088 | 0.00484358 |
| 283078 | MKX | mohawk homeobox | -1.648518088 | 0.00175462 |
| 4907 | NT5E | 5'-nucleotidase ecto | -1.647123142 | 0.00175462 |
| 27074 | LAMP3 | lysosomal associated membrane protein 3 | -1.599374648 | 0.02668486 |
| 2674 | GFRA1 | GDNF family receptor alpha 1 | -1.587016082 | 0.01158811 |
| 2555 | GABRA2 | gamma-aminobutyric acid type A receptor alpha2 subunit | -1.573438293 | 0.00410634 |
| 2118 | ETV4 | ETS variant 4 | -1.522098082 | 0.0096345 |
| 340120 | ANKRD34B | ankyrin repeat domain 34B | -1.493051401 | 0.0412427 |
| 164832 | LONRF2 | LON peptidase N-terminal domain and ring finger 2 | -1.462472668 | 0.00942377 |
| 994 | CDC25B | cell division cycle 25B | -1.455420892 | 0.00244396 |
| 467 | ATF3 | activating transcription factor 3 | -1.406916723 | 0.01225316 |
| 10398 | MYL9 | myosin light chain 9 | -1.384477122 | 0.03684424 |
| 80329 | ULBP1 | UL16 binding protein 1 | -1.378068251 | 0.04777524 |
| 255488 | RNF144B | ring finger protein 144B | -1.363722826 | 0.01457651 |
| 389493 | NUPR2 | nuclear protein 2, transcriptional regulator | -1.354324527 | 0.04899974 |
| 2335 | FN1 | fibronectin 1 | -1.351722241 | 0.0944861 |
| 161823 | ADAL | adenosine deaminase-like | -1.334270991 | 0.01781217 |
| 389421 | LIN28B | lin-28 homolog B | -1.328891774 | 0.087099 |
| 2039 | DMTN | dematin actin binding protein | -1.327696291 | 0.00633027 |
| 9518 | GDF15 | growth differentiation factor 15 | -1.312379701 | 0.05037868 |
| 827 | CAPN6 | calpain 6 | -1.288998697 | 0.04743888 |
| 2872 | MKNK2 | MAP kinase interacting serine/threonine kinase 2 | -1.263730224 | 0.00564086 |
| 54899 | PXK | PX domain containing serine/threonine kinase like | -1.25568287 | 0.00661123 |
| 4199 | ME1 | malic enzyme 1, NADP(+)-dependent, cytosolic | -1.253259726 | 0.01628247 |
| 1054 | CEBPG | CCAAT/enhancer binding protein gamma | -1.241648562 | 0.03235839 |
| 4862 | NPAS2 | neuronal PAS domain protein 2 | -1.237863134 | 0.01225316 |
| 144501 | KRT80 | keratin 80 | -1.237363857 | 0.06084381 |
| 23135 | KDM6B | lysine demethylase 6B | -1.236581542 | 0.02763628 |
| 367 | AR | androgen receptor | -1.22503311 | 0.04405657 |
| 345651 | ACTBL2 | actin, beta-like 2 | -1.220584169 | 0.04674781 |
| 2931 | GSK3A | glycogen synthase kinase 3 alpha | -1.187230768 | 0.01420318 |
| 54742 | LY6K | lymphocyte antigen 6 complex, locus K | -1.177847045 | 0.01756818 |
| 2296 | FOXC1 | forkhead box C1 | -1.177816913 | 0.02378993 |
| 4849 | CNOT3 | CCR4-NOT transcription complex subunit 3 | -1.175966502 | 0.07755758 |
| 113026 | PLCD3 | phospholipase C delta 3 | -1.167897648 | 0.05037868 |
| 6536 | SLC6A9 | solute carrier family 6 member 9 | -1.163135445 | 0.06989556 |
| 30844 | EHD4 | EH domain containing 4 | -1.153228731 | 0.01158811 |
| 6769 | STAC | SH3 and cysteine rich domain | -1.147918615 | 0.03334146 |
| 9830 | TRIM14 | tripartite motif containing 14 | -1.14527533 | 0.0464794 |
| 56648 | EIF5A2 | eukaryotic translation initiation factor 5A2 | -1.145151643 | 0.05130111 |
| 9760 | TOX | thymocyte selection associated high mobility group box | -1.143373883 | 0.06791837 |
| 2000 | ELF4 | E74 like ETS transcription factor 4 | -1.140824028 | 0.06403603 |
| 65059 | RAPH1 | Ras association (RalGDS/AF-6) and pleckstrin homology domains 1 | -1.132746826 | 0.01900394 |
| 56907 | SPIRE1 | spire type actin nucleation factor 1 | -1.096413743 | 0.08184589 |
| 100873765 | RNU6-57P | RNA, U6 small nuclear 57, pseudogene | -1.095161 | 0.0944861 |
| 396 | ARHGDIA | Rho GDP dissociation inhibitor (GDI) alpha | -1.092410676 | 0.01560692 |
| 644172 | LOC644172 | mitogen-activated protein kinase 8 interacting protein 1 pseudogene | -1.09110629 | 0.07610795 |
| 154 | ADRB2 | adrenoceptor beta 2 | -1.085064454 | 0.01628247 |
| 6756 | SSX1 | synovial sarcoma, X breakpoint 1 | -1.08050026 | 0.06789671 |
| 25946 | ZNF385A | zinc finger protein 385A | -1.075518503 | 0.05699118 |
| 2081 | ERN1 | endoplasmic reticulum to nucleus signaling 1 | -1.070016223 | 0.01950308 |
| 1728 | NQO1 | NAD(P)H dehydrogenase, quinone 1 | -1.063826656 | 0.01797073 |
| 84777 | DLGAP1-AS2 | DLGAP1 antisense RNA 2 | -1.062567652 | 0.08523757 |
| 23057 | NMNAT2 | nicotinamide nucleotide adenylyltransferase 2 | -1.060291562 | 0.04877887 |
| 4088 | SMAD3 | SMAD family member 3 | -1.058700953 | 0.00760654 |
| 92154 | MTSS1L | metastasis suppressor 1-like | -1.05599671 | 0.01603362 |
| 124801 | LSM12 | LSM12 homolog | -1.048860817 | 0.08404296 |
| 55214 | P3H2 | prolyl 3-hydroxylase 2 | -1.047337453 | 0.07610795 |
| 7703 | PCGF2 | polycomb group ring finger 2 | -1.046135509 | 0.03334146 |
| 5106 | PCK2 | phosphoenolpyruvate carboxykinase 2, mitochondrial | -1.041359724 | 0.04210457 |
| 7357 | UGCG | UDP-glucose ceramide glucosyltransferase | -1.035556647 | 0.01004408 |
| 5326 | PLAGL2 | PLAG1 like zinc finger 2 | -1.032616227 | 0.0155577 |
| 6455 | SH3GL1 | SH3-domain GRB2-like 1 | -1.030468894 | 0.06773403 |
| 63894 | VIPAS39 | VPS33B interacting protein, apical-basolateral polarity regulator, spe-39 homolog | -1.029071073 | 0.01797073 |
| 81796 | SLCO5A1 | solute carrier organic anion transporter family member 5A1 | -1.026095647 | 0.01628247 |
| 127829 | ARL8A | ADP ribosylation factor like GTPase 8A | -1.024532968 | 0.01253987 |
| 25792 | CIZ1 | CDKN1A interacting zinc finger protein 1 | -1.019422738 | 0.0118484 |
| 283149 | BCL9L | B-cell CLL/lymphoma 9-like | -1.01756129 | 0.04796445 |
| 56731 | SLC2A4RG | SLC2A4 regulator | -1.016451881 | 0.01225316 |
| 100133172 | FAM66A | family with sequence similarity 66 member A | -1.012670296 | 0.0412427 |
| 55311 | ZNF444 | zinc finger protein 444 | -1.004095266 | 0.06123948 |
| 389856 | USP27X | ubiquitin specific peptidase 27, X-linked | -0.998665248 | 0.07466991 |
| 57478 | USP31 | ubiquitin specific peptidase 31 | -0.997594714 | 0.03248308 |
| 10979 | FERMT2 | fermitin family member 2 | -0.993779911 | 0.03779754 |
| 23034 | SAMD4A | sterile alpha motif domain containing 4A | -0.989592586 | 0.05169334 |
| 112939 | NACC1 | nucleus accumbens associated 1 | -0.983628534 | 0.0412427 |
| 6815 | STYX | serine/threonine/tyrosine interacting protein | -0.980197654 | 0.01305734 |
| 203197 | C9orf91 | chromosome 9 open reading frame 91 | -0.972797899 | 0.01420318 |
| 645700 | ZNF890P | zinc finger protein 890, pseudogene | -0.970941991 | 0.0609728 |
| 91369 | ANKRD40 | ankyrin repeat domain 40 | -0.970804091 | 0.05699256 |
| 8976 | WASL | Wiskott-Aldrich syndrome-like | -0.969798117 | 0.02548913 |
| 9991 | PTBP3 | polypyrimidine tract binding protein 3 | -0.968107916 | 0.02559856 |
| 54877 | ZCCHC2 | zinc finger CCHC-type containing 2 | -0.96642852 | 0.00633027 |
| 126868 | MAB21L3 | mab-21-like 3 (C. elegans) | -0.964943378 | 0.05648737 |
| 116372 | LYPD1 | LY6/PLAUR domain containing 1 | -0.961744729 | 0.04743888 |
| 146664 | MGAT5B | mannosyl (alpha-1,6-)-glycoprotein beta-1,6-N-acetyl-glucosaminyltransferase, isozyme B | -0.953319363 | 0.0199973 |
| 9672 | SDC3 | syndecan 3 | -0.947542925 | 0.03248308 |
| 4771 | NF2 | neurofibromin 2 (merlin) | -0.946069815 | 0.01712341 |
| 29979 | UBQLN1 | ubiquilin 1 | -0.928198604 | 0.05436009 |
| 79890 | RIN3 | Ras and Rab interactor 3 | -0.928156534 | 0.04674781 |
| 89853 | MVB12B | multivesicular body subunit 12B | -0.926979124 | 0.01253987 |
| 57510 | XPO5 | exportin 5 | -0.926178019 | 0.01158811 |
| 1958 | EGR1 | early growth response 1 | -0.924940207 | 0.03638031 |
| 399665 | FAM102A | family with sequence similarity 102 member A | -0.924061751 | 0.03937365 |
| 64864 | RFX7 | regulatory factor X7 | -0.920941994 | 0.01158811 |
| 83439 | TCF7L1 | transcription factor 7 like 1 | -0.918406499 | 0.03506031 |
| 63874 | ABHD4 | abhydrolase domain containing 4 | -0.913458655 | 0.087099 |
| 7791 | ZYX | zyxin | -0.90717667 | 0.03334146 |
| 27092 | CACNG4 | calcium voltage-gated channel auxiliary subunit gamma 4 | -0.905031998 | 0.03722192 |
| 11221 | DUSP10 | dual specificity phosphatase 10 | -0.901152587 | 0.03454101 |
| 55329 | MNS1 | meiosis specific nuclear structural 1 | 0.905173005 | 0.05037868 |
| 84898 | PLXDC2 | plexin domain containing 2 | 0.906325544 | 0.01420318 |
| 677810 | SNORA26 | small nucleolar RNA, H/ACA box 26 | 0.907500948 | 0.09151182 |
| 2896 | GRN | granulin | 0.911008918 | 0.03283646 |
| 85495 | RPPH1 | ribonuclease P RNA component H1 | 0.911551052 | 0.01540301 |
| 9510 | ADAMTS1 | ADAM metallopeptidase with thrombospondin type 1 motif 1 | 0.91269036 | 0.07553921 |
| 10956 | OS9 | osteosarcoma amplified 9, endoplasmic reticulum lectin | 0.917340578 | 0.01305734 |
| 976 | ADGRE5 | adhesion G protein-coupled receptor E5 | 0.926186908 | 0.04928186 |
| 6558 | SLC12A2 | solute carrier family 12 member 2 | 0.929787647 | 0.05382065 |
| 55064 | SPATA6L | spermatogenesis associated 6 like | 0.934634396 | 0.01603362 |
| 7072 | TIA1 | TIA1 cytotoxic granule-associated RNA binding protein | 0.934856299 | 0.02590557 |
| 100506233 | RAB30-AS1 | RAB30 antisense RNA 1 (head to head) | 0.935109662 | 0.01569543 |
| 56649 | TMPRSS4 | transmembrane protease, serine 4 | 0.936693387 | 0.06900342 |
| 5144 | PDE4D | phosphodiesterase 4D | 0.938397639 | 0.06642155 |
| 121227 | LRIG3 | leucine-rich repeats and immunoglobulin like domains 3 | 0.939756633 | 0.03990694 |
| 79915 | ATAD5 | ATPase family, AAA domain containing 5 | 0.941464144 | 0.02966508 |
| 378938 | MALAT1 | metastasis associated lung adenocarcinoma transcript 1 (non-protein coding) | 0.941475206 | 0.04217591 |
| 145864 | HAPLN3 | hyaluronan and proteoglycan link protein 3 | 0.942225997 | 0.09344376 |
| 546 | ATRX | alpha thalassemia/mental retardation syndrome X-linked | 0.95095626 | 0.06376815 |
| 3150 | HMGN1 | high mobility group nucleosome binding domain 1 | 0.957848546 | 0.02299024 |
| 51279 | C1RL | complement C1r subcomponent like | 0.958606446 | 0.0118484 |
| 7083 | TK1 | thymidine kinase 1, soluble | 0.959593616 | 0.06376815 |
| 100506451 | RASSF8-AS1 | RASSF8 atnisense RNA 1 | 0.963501772 | 0.01640007 |
| 100873774 | RNU6-71P | RNA, U6 small nuclear 71, pseudogene | 0.965829609 | 0.06030077 |
| 290 | ANPEP | alanyl aminopeptidase, membrane | 0.970373042 | 0.06644095 |
| 10406 | WFDC2 | WAP four-disulfide core domain 2 | 0.972520975 | 0.09428695 |
| 692209 | SNORD92 | small nucleolar RNA, C/D box 92 | 0.974075714 | 0.06907875 |
| 55273 | TMEM100 | transmembrane protein 100 | 0.976391256 | 0.01560692 |
| 200162 | SPAG17 | sperm associated antigen 17 | 0.978457267 | 0.02149137 |
| 94161 | SNORD46 | small nucleolar RNA, C/D box 46 | 0.98434441 | 0.01420318 |
| 256356 | GK5 | glycerol kinase 5 (putative) | 0.988752276 | 0.05355547 |
| 100379345 | MIR181A2HG | MIR181A2 host gene | 0.989739508 | 0.02478766 |
| 23461 | ABCA5 | ATP binding cassette subfamily A member 5 | 0.989819695 | 0.03374872 |
| 9068 | ANGPTL1 | angiopoietin like 1 | 0.990302311 | 0.08749785 |
| 387036 | GUSBP2 | glucuronidase, beta pseudogene 2 | 0.990373823 | 0.02504743 |
| 692195 | SNORD75 | small nucleolar RNA, C/D box 75 | 0.990448111 | 0.01569543 |
| 143279 | HECTD2 | HECT domain E3 ubiquitin protein ligase 2 | 0.992113516 | 0.04550794 |
| 1191 | CLU | clusterin | 0.992213781 | 0.02983682 |
| 56154 | TEX15 | testis expressed 15 | 0.995805203 | 0.06950085 |
| 406885 | MIRLET7C | microRNA let-7c | 0.996274555 | 0.02478766 |
| 9489 | PGS1 | phosphatidylglycerophosphate synthase 1 | 0.996482316 | 0.02763628 |
| 100131131 | LOC100131131 | AHPA9419 | 0.999262825 | 0.06100747 |
| 100505687 | LINC00888 | long intergenic non-protein coding RNA 888 | 0.999490159 | 0.04302163 |
| 10232 | MSLN | mesothelin | 1.001272835 | 0.01628247 |
| 677845 | SNORA79 | small nucleolar RNA, H/ACA box 79 | 1.005827756 | 0.02711563 |
| 23566 | LPAR3 | lysophosphatidic acid receptor 3 | 1.010956509 | 0.07466991 |
| 790953 | TSL | testis-expressed, seven-twelve, leukemia | 1.011478237 | 0.09944443 |
| 6077 | SNORD13P2 | small nucleolar RNA, C/D box 13 pseudogene 2 | 1.021367108 | 0.04830056 |
| 442891 | MIR135B | microRNA 135b | 1.023033011 | 0.00633027 |
| 100616144 | MIR548AN | microRNA 548an | 1.030813586 | 0.05463585 |
| 6171 | RPL41 | ribosomal protein L41 | 1.032207227 | 0.03708232 |
| 26812 | SNORD37 | small nucleolar RNA, C/D box 37 | 1.034985629 | 0.02763628 |
| 407036 | MIR32 | microRNA 32 | 1.041073496 | 0.04330613 |
| 151613 | TTC14 | tetratricopeptide repeat domain 14 | 1.04662868 | 0.01147176 |
| 230 | ALDOC | aldolase, fructose-bisphosphate C | 1.047698603 | 0.06505559 |
| 8335 | HIST1H2AB | histone cluster 1, H2ab | 1.050749604 | 0.06773403 |
| 5265 | SERPINA1 | serpin peptidase inhibitor, clade A (alpha-1 antiproteinase, antitrypsin), member 1 | 1.052566782 | 0.03248308 |
| 445582 | POTEE | POTE ankyrin domain family member E | 1.053889641 | 0.05377509 |
| 692225 | SNORD94 | small nucleolar RNA, C/D box 94 | 1.054306972 | 0.03708232 |
| 1351 | COX8A | cytochrome c oxidase subunit 8A | 1.055998983 | 0.07705903 |
| 401585 | LOC401585 | uncharacterized LOC401585 | 1.059485193 | 0.02966508 |
| 100616299 | MIR4450 | microRNA 4450 | 1.059701422 | 0.09151182 |
| 55 | ACPP | acid phosphatase, prostate | 1.068715047 | 0.03506031 |
| 116937 | SNORD83A | small nucleolar RNA, C/D box 83A | 1.069466527 | 0.04728391 |
| 2139 | EYA2 | EYA transcriptional coactivator and phosphatase 2 | 1.069866602 | 0.04855936 |
| 3791 | KDR | kinase insert domain receptor | 1.072070189 | 0.02191849 |
| 100033427 | SNORD116-15 | small nucleolar RNA, C/D box 116-15 | 1.072777541 | 0.06030077 |
| 728621 | CCDC30 | coiled-coil domain containing 30 | 1.075137458 | 0.04397617 |
| 1062 | CENPE | centromere protein E | 1.076176496 | 0.02076927 |
| 8701 | DNAH11 | dynein axonemal heavy chain 11 | 1.076607021 | 0.02605765 |
| 54491 | FAM105A | family with sequence similarity 105 member A | 1.089637338 | 0.07648411 |
| 9619 | ABCG1 | ATP binding cassette subfamily G member 1 | 1.091603091 | 0.04397617 |
| 388815 | MIR99AHG | mir-99a-let-7c cluster host gene | 1.098810563 | 0.02020568 |
| 26785 | SNORD63 | small nucleolar RNA, C/D box 63 | 1.099101998 | 0.0944861 |
| 84440 | RAB11FIP4 | RAB11 family interacting protein 4 (class II) | 1.099160389 | 0.02828493 |
| 9585 | KIF20B | kinesin family member 20B | 1.105738567 | 0.03768917 |
| 50840 | TAS2R14 | taste 2 receptor member 14 | 1.11328805 | 0.01225316 |
| 259266 | ASPM | abnormal spindle microtubule assembly | 1.115844969 | 0.03248308 |
| 2981 | GUCA2B | guanylate cyclase activator 2B | 1.125272096 | 0.03998617 |
| 692107 | SNORD66 | small nucleolar RNA, C/D box 66 | 1.133769998 | 0.06414385 |
| 3485 | IGFBP2 | insulin like growth factor binding protein 2 | 1.134941847 | 0.02058007 |
| 79949 | PLEKHS1 | pleckstrin homology domain containing S1 | 1.135319403 | 0.0944861 |
| 6513 | SLC2A1 | solute carrier family 2 member 1 | 1.147702108 | 0.00240634 |
| 54082 | TSPEAR-AS1 | TSPEAR antisense RNA 1 | 1.148493234 | 0.01569543 |
| 677826 | SNORA3B | small nucleolar RNA, H/ACA box 3B | 1.149352224 | 0.04631721 |
| 100506123 | LOC100506123 | uncharacterized LOC100506123 | 1.154485915 | 0.06484681 |
| 8991 | SELENBP1 | selenium binding protein 1 | 1.159496431 | 0.00633027 |
| 5797 | PTPRM | protein tyrosine phosphatase, receptor type M | 1.177111959 | 0.06915117 |
| 692149 | SCARNA14 | small Cajal body-specific RNA 14 | 1.184692648 | 0.02813024 |
| 693148 | MIR563 | microRNA 563 | 1.186536655 | 0.06446851 |
| 677774 | SCARNA1 | small Cajal body-specific RNA 1 | 1.19046827 | 0.03235839 |
| 100505727 | LOC100505727 | uncharacterized LOC100505727 | 1.191873458 | 0.04877887 |
| 100500854 | MIR3671 | microRNA 3671 | 1.191908361 | 0.07755758 |
| 7178 | TPT1 | tumor protein, translationally-controlled 1 | 1.194949918 | 0.00665116 |
| 654321 | SNORA75 | small nucleolar RNA, H/ACA box 75 | 1.198476413 | 0.02157794 |
| 100124535 | SNORA36C | small nucleolar RNA, H/ACA box 36C | 1.203916662 | 0.06267193 |
| 8759 | ADAM1A | ADAM metallopeptidase domain 1A (pseudogene) | 1.209475827 | 0.02073301 |
| 677763 | SCARNA21 | small Cajal body-specific RNA 21 | 1.209910484 | 0.0676994 |
| 677840 | SNORA71D | small nucleolar RNA, H/ACA box 71D | 1.219040842 | 0.087099 |
| 2804 | GOLGB1 | golgin B1 | 1.221591373 | 0.00513322 |
| 692058 | SNORD11 | small nucleolar RNA, C/D box 11 | 1.224917557 | 0.09151182 |
| 692111 | SNORD71 | small nucleolar RNA, C/D box 71 | 1.227195919 | 0.01569543 |
| 692229 | SNORD105 | small nucleolar RNA, C/D box 105 | 1.232492894 | 0.00399429 |
| 6646 | SOAT1 | sterol O-acyltransferase 1 | 1.237926107 | 0.00410634 |
| 91975 | ZNF300 | zinc finger protein 300 | 1.240251172 | 0.04397617 |
| 654322 | SNORA13 | small nucleolar RNA, H/ACA box 13 | 1.243140975 | 0.04063827 |
| 100033413 | SNORD116-1 | small nucleolar RNA, C/D box 116-1 | 1.252678846 | 0.0609728 |
| 100033438 | SNORD116-26 | small nucleolar RNA, C/D box 116-26 | 1.258074117 | 0.05037868 |
| 677775 | SCARNA5 | small Cajal body-specific RNA 5 | 1.259610688 | 0.01158811 |
| 677767 | SCARNA7 | small Cajal body-specific RNA 7 | 1.265824515 | 0.01569543 |
| 658 | BMPR1B | bone morphogenetic protein receptor type 1B | 1.274135953 | 0.00373959 |
| 677842 | SNORA50C | small nucleolar RNA, H/ACA box 50C | 1.277529272 | 0.02668486 |
| 29100 | TMEM208 | transmembrane protein 208 | 1.278380446 | 0.03684424 |
| 7919 | DDX39B | DEAD-box helicase 39B | 1.288505785 | 0.06376815 |
| 10142 | AKAP9 | A-kinase anchoring protein 9 | 1.302587725 | 0.00395068 |
| 677801 | SNORA14A | small nucleolar RNA, H/ACA box 14A | 1.304519237 | 0.07658987 |
| 100113391 | SNORD126 | small nucleolar RNA, C/D box 126 | 1.307433025 | 0.02986914 |
| 100151683 | RNU4ATAC | RNA, U4atac small nuclear (U12-dependent splicing) | 1.308170296 | 0.01529192 |
| 79365 | BHLHE41 | basic helix-loop-helix family member e41 | 1.314383417 | 0.00537783 |
| 2013 | EMP2 | epithelial membrane protein 2 | 1.314537307 | 0.00674016 |
| 677769 | SCARNA17 | small Cajal body-specific RNA 17 | 1.319844085 | 0.00399429 |
| 677772 | SCARNA6 | small Cajal body-specific RNA 6 | 1.322669569 | 0.02763628 |
| 677828 | SNORA47 | small nucleolar RNA, H/ACA box 47 | 1.326764412 | 0.02228275 |
| 100422909 | MIR4295 | microRNA 4295 | 1.332515952 | 0.0294947 |
| 28 | ABO | ABO blood group (transferase A, alpha 1-3-N-acetylgalactosaminyltransferase; transferase B, alpha 1-3-galactosyltransferase) | 1.340727498 | 0.06330012 |
| 677834 | SNORA55 | small nucleolar RNA, H/ACA box 55 | 1.340887945 | 0.0174397 |
| 23515 | MORC3 | MORC family CW-type zinc finger 3 | 1.342234957 | 0.02106368 |
| 245711 | SPDYA | speedy/RINGO cell cycle regulator family member A | 1.360601308 | 0.02504743 |
| 100422864 | MIR544B | microRNA 544b | 1.362035013 | 0.03248308 |
| 100033435 | SNORD116-24 | small nucleolar RNA, C/D box 116-24 | 1.375287788 | 0.00273842 |
| 26822 | SNORD14A | small nucleolar RNA, C/D box 14A | 1.380373264 | 0.00399429 |
| 100507012 | BMPR1B-AS1 | BMPR1B antisense RNA 1 (head to head) | 1.387639031 | 0.02192194 |
| 100113379 | SNORD121A | small nucleolar RNA, C/D box 121A | 1.391287382 | 0.044511 |
| 677830 | SNORA50A | small nucleolar RNA, H/ACA box 50A | 1.391417088 | 0.00270503 |
| 677773 | SCARNA23 | small Cajal body-specific RNA 23 | 1.391764919 | 0.087099 |
| 677793 | SNORA2A | small nucleolar RNA, H/ACA box 2A | 1.428672722 | 0.04330613 |
| 319103 | SNORD8 | small nucleolar RNA, C/D box 8 | 1.434691933 | 0.01464013 |
| 388677 | NOTCH2NL | notch 2 N-terminal like | 1.440753202 | 0.02828493 |
| 5163 | PDK1 | pyruvate dehydrogenase kinase 1 | 1.446266323 | 0.00148773 |
| 100158262 | SCARNA9L | small Cajal body-specific RNA 9-like | 1.465874824 | 0.00157675 |
| 25840 | METTL7A | methyltransferase like 7A | 1.485936601 | 0.01603362 |
| 100379132 | SNORA70G | small nucleolar RNA, H/ACA box 70G | 1.49373169 | 0.00550924 |
| 100129233 | LOC100129233 | uncharacterized LOC100129233 | 1.506138861 | 0.01420318 |
| 26783 | SNORA65 | small nucleolar RNA, H/ACA box 65 | 1.508772705 | 0.01569543 |
| 6507 | SLC1A3 | solute carrier family 1 member 3 | 1.515492893 | 0.09922847 |
| 5892 | RAD51D | RAD51 paralog D | 1.529063312 | 0.07577869 |
| 677800 | SNORA12 | small nucleolar RNA, H/ACA box 12 | 1.540583352 | 0.00084076 |
| 406949 | MIR15B | microRNA 15b | 1.563458227 | 0.0287958 |
| 170689 | ADAMTS15 | ADAM metallopeptidase with thrombospondin type 1 motif 15 | 1.583918832 | 0.01489335 |
| 406985 | MIR200C | microRNA 200c | 1.587203326 | 0.0294947 |
| 677771 | SCARNA4 | small Cajal body-specific RNA 4 | 1.596946103 | 0.00161275 |
| 692086 | SNORD17 | small nucleolar RNA, C/D box 17 | 1.6074462 | 0.0080529 |
| 100124536 | SNORA38B | small nucleolar RNA, H/ACA box 38B | 1.626201574 | 0.09096278 |
| 693229 | MIR644A | microRNA 644a | 1.638591746 | 0.0273193 |
| 100113384 | SNORD123 | small nucleolar RNA, C/D box 123 | 1.679318383 | 0.03329589 |
| 56477 | CCL28 | C-C motif chemokine ligand 28 | 1.684765795 | 0.00410634 |
| 26810 | SNORD41 | small nucleolar RNA, C/D box 41 | 1.690645147 | 0.00399429 |
| 54855 | FAM46C | family with sequence similarity 46 member C | 1.695916914 | 0.00484358 |
| 692233 | SNORD117 | small nucleolar RNA, C/D box 117 | 1.696102489 | 0.0096345 |
| 4118 | MAL | mal T-cell differentiation protein | 1.699801561 | 0.07039261 |
| 100126299 | VTRNA2-1 | vault RNA 2-1 | 1.707485573 | 0.03454101 |
| 677681 | SCARNA20 | small Cajal body-specific RNA 20 | 1.713004634 | 0.02763628 |
| 353189 | SLCO4C1 | solute carrier organic anion transporter family member 4C1 | 1.727061147 | 0.00357025 |
| 5033 | P4HA1 | prolyl 4-hydroxylase subunit alpha 1 | 1.743839453 | 0.00084076 |
| 26832 | RNU5B-1 | RNA, U5B small nuclear 1 | 1.751050835 | 0.07466991 |
| 692148 | SCARNA10 | small Cajal body-specific RNA 10 | 1.753569273 | 0.00525035 |
| 10388 | SYCP2 | synaptonemal complex protein 2 | 1.760565863 | 0.09077191 |
| 1356 | CP | ceruloplasmin (ferroxidase) | 1.769252419 | 0.02763628 |
| 8483 | CILP | cartilage intermediate layer protein | 1.775627877 | 0.06100747 |
| 677820 | SNORA38 | small nucleolar RNA, H/ACA box 38 | 1.779744648 | 0.01431086 |
| 692207 | SNORD91A | small nucleolar RNA, C/D box 91A | 1.779773724 | 0.087099 |
| 677770 | SCARNA22 | small Cajal body-specific RNA 22 | 1.780607574 | 0.00564086 |
| 5087 | PBX1 | pre-B-cell leukemia homeobox 1 | 1.783943674 | 0.01628247 |
| 664 | BNIP3 | BCL2/adenovirus E1B 19kDa interacting protein 3 | 1.792636844 | 0.00122419 |
| 677819 | SNORA37 | small nucleolar RNA, H/ACA box 37 | 1.844953423 | 0.02183078 |
| 26824 | RNU11 | RNA, U11 small nuclear | 1.863411074 | 0.0294947 |
| 594839 | SNORA33 | small nucleolar RNA, H/ACA box 33 | 1.868444173 | 0.01628247 |
| 677802 | SNORA14B | small nucleolar RNA, H/ACA box 14B | 1.877599781 | 0.00157141 |
| 100313770 | MIR548K | microRNA 548k | 1.895222131 | 0.00244396 |
| 100313771 | MIR548F2 | microRNA 548f-2 | 1.915963313 | 0.0155577 |
| 677837 | SNORA60 | small nucleolar RNA, H/ACA box 60 | 1.91824322 | 0.06765115 |
| 692214 | SNORD111 | small nucleolar RNA, C/D box 111 | 1.919984312 | 0.00633027 |
| 692110 | SNORD70 | small nucleolar RNA, C/D box 70 | 1.946664044 | 0.0287958 |
| 677827 | SNORA46 | small nucleolar RNA, H/ACA box 46 | 1.972660821 | 0.01158811 |
| 56663 | VTRNA1-2 | vault RNA 1-2 | 2.127765307 | 0.0287958 |
| 692057 | SNORD12 | small nucleolar RNA, C/D box 12 | 2.224131564 | 0.00890057 |
| 1368 | CPM | carboxypeptidase M | 2.282703827 | 0.03466562 |
| 780853 | SNORD3C | small nucleolar RNA, C/D box 3C | 2.319353927 | 0.00084076 |
| 677823 | SNORA80E | small nucleolar RNA, H/ACA box 80E | 2.364383307 | 0.0287958 |
| 26765 | SNORD12C | small nucleolar RNA, C/D box 12C | 2.555545973 | 0.00548995 |
| 677776 | SCARNA8 | small Cajal body-specific RNA 8 | 2.564468548 | 0.00221467 |
| 100113393 | SNORD12B | small nucleolar RNA, C/D box 12B | 2.619701023 | 0.00157141 |
