# Supplementary material for: The epigenetic factor BORIS (CTCFL) controls the androgen receptor regulatory network in ovarian cancer
Source: Oncogenesis. 2019 Aug 12;8(8):41. doi: 10.1038/s41389-019-0150-2 (PMC6690894; doi:10.1038/s41389-019-0150-2)
Supplement: Supplementary file 8 — Supplementary Table 2 [file 41389_2019_150_MOESM8_ESM.docx]

Supplementary Table 2. Differentially expressed genes identified in BORIS Knockout cells versus Control

| ENTREZID | SYMBOL | GENENAME | logFC | adj.P.Val |
| --- | --- | --- | --- | --- |
| 63874 | ABHD4 | abhydrolase domain containing 4 | -0.956354781 | 0.080638403 |
| 25 | ABL1 | ABL proto-oncogene 1, non-receptor tyrosine kinase | -1.075564999 | 0.050136508 |
| 55 | ACPP | acid phosphatase, prostate | 1.101021041 | 0.038139938 |
| 345651 | ACTBL2 | actin, beta-like 2 | 1.111288972 | 0.072038558 |
| 100 | ADA | adenosine deaminase | 1.669550401 | 0.061433637 |
| 161823 | ADAL | adenosine deaminase-like | -1.483460656 | 0.016776584 |
| 8759 | ADAM1A | ADAM metallopeptidase domain 1A (pseudogene) | 1.106787846 | 0.034149818 |
| 976 | ADGRE5 | adhesion G protein-coupled receptor E5 | 1.033496222 | 0.040483698 |
| 23287 | AGTPBP1 | ATP/GTP binding protein 1 | -0.977611945 | 0.024593471 |
| 196 | AHR | aryl hydrocarbon receptor | -1.05894309 | 0.009312563 |
| 230 | ALDOC | aldolase, fructose-bisphosphate C | 1.277936264 | 0.038518139 |
| 121642 | ALKBH2 | alkB homolog 2, alpha-ketoglutarate-dependent dioxygenase | 1.284093621 | 0.053134809 |
| 56172 | ANKH | ANKH inorganic pyrophosphate transport regulator | -0.979708793 | 0.053134809 |
| 203859 | ANO5 | anoctamin 5 | -1.555240916 | 0.05253635 |
| 353 | APRT | adenine phosphoribosyltransferase | 1.184462339 | 0.061911697 |
| 367 | AR | androgen receptor | -1.760366479 | 0.015828172 |
| 374 | AREG | amphiregulin | 1.939062626 | 0.024116259 |
| 57221 | ARFGEF3 | ARFGEF family member 3 | -0.99977538 | 0.040204907 |
| 23092 | ARHGAP26 | Rho GTPase activating protein 26 | -1.070418649 | 0.01987844 |
| 396 | ARHGDIA | Rho GDP dissociation inhibitor (GDI) alpha | -1.003169469 | 0.026014755 |
| 54622 | ARL15 | ADP ribosylation factor like GTPase 15 | 0.966142419 | 0.057549239 |
| 23204 | ARL6IP1 | ADP ribosylation factor like GTPase 6 interacting protein 1 | 1.076331473 | 0.01537599 |
| 467 | ATF3 | activating transcription factor 3 | -1.053768546 | 0.039089657 |
| 491 | ATP2B2 | ATPase plasma membrane Ca2+ transporting 2 | 1.050781611 | 0.058044611 |
| 516 | ATP5G1 | ATP synthase, H+ transporting, mitochondrial Fo complex subunit C1 (subunit 9) | 0.924608574 | 0.033252629 |
| 8708 | B3GALT1 | Beta-1,3-galactosyltransferase 1 | -1.821775599 | 0.019424115 |
| 10134 | BCAP31 | B-cell receptor-associated protein 31 | 1.175673517 | 0.0075272 |
| 53335 | BCL11A | B-cell CLL/lymphoma 11A | -1.687900869 | 0.074199983 |
| 283149 | BCL9L | B-cell CLL/lymphoma 9-like | -0.952093687 | 0.067855301 |
| 100507012 | BMPR1B-AS1 | BMPR1B antisense RNA 1 (head to head) | 0.966765023 | 0.085419279 |
| 100506674 | BRCAT54 | breast cancer-associated transcript 54 | 0.957370438 | 0.021349686 |
| 684 | BST2 | bone marrow stromal cell antigen 2 | 2.326818991 | 0.009814848 |
| 113246 | C12orf57 | chromosome 12 open reading frame 57 | 1.039897117 | 0.082687849 |
| 126526 | C19orf47 | chromosome 19 open reading frame 47 | -1.45821262 | 0.083211481 |
| 728819 | C1GALT1C1L | C1GALT1-specific chaperone 1 like | 0.930196944 | 0.093960605 |
| 643988 | C1orf233 | chromosome 1 open reading frame 233 | -1.113409955 | 0.033252629 |
| 716 | C1S | complement component 1, s subcomponent | 1.133916777 | 0.057395772 |
| 56892 | C8orf4 | chromosome 8 open reading frame 4 | 2.114055603 | 0.029123846 |
| 401466 | C8orf59 | chromosome 8 open reading frame 59 | 1.162675334 | 0.024078668 |
| 827 | CAPN6 | calpain 6 | -1.550110341 | 0.030773122 |
| 343099 | CCDC18 | coiled-coil domain containing 18 | 0.986168016 | 0.014511128 |
| 91057 | CCDC34 | coiled-coil domain containing 34 | 1.162270022 | 0.099715992 |
| 56477 | CCL28 | C-C motif chemokine ligand 28 | 1.368578288 | 0.01537599 |
| 958 | CD40 | CD40 molecule | 0.957329343 | 0.024663673 |
| 978 | CDA | cytidine deaminase | 1.195000136 | 0.098834032 |
| 994 | CDC25B | cell division cycle 25B | -1.223461499 | 0.009733369 |
| 5218 | CDK14 | cyclin-dependent kinase 14 | -1.169898918 | 0.00619556 |
| 1021 | CDK6 | cyclin-dependent kinase 6 | -1.048902686 | 0.034149818 |
| 1038 | CDR1 | cerebellar degeneration related protein 1 | -2.740437354 | 0.02693268 |
| 256223 | CDRT15L2 | CMT1A duplicated region transcript 15-like 2 | 1.459913901 | 0.098023177 |
| 1054 | CEBPG | CCAAT/enhancer binding protein gamma | -1.189951082 | 0.043344501 |
| 1062 | CENPE | centromere protein E | 1.102113537 | 0.024663673 |
| 64946 | CENPH | centromere protein H | 0.925746077 | 0.0799589 |
| 2491 | CENPI | centromere protein I | 1.378438688 | 0.037819952 |
| 22832 | CEP162 | centrosomal protein 162kDa | 0.949947103 | 0.038139938 |
| 1070 | CETN3 | centrin 3 | 1.010751309 | 0.076886469 |
| 10519 | CIB1 | calcium and integrin binding 1 | 0.98277907 | 0.052097556 |
| 25792 | CIZ1 | CDKN1A interacting zinc finger protein 1 | -1.060177317 | 0.01382131 |
| 9071 | CLDN10 | claudin 10 | 1.49037637 | 0.029123846 |
| 64084 | CLSTN2 | calsyntenin 2 | -1.895737794 | 0.017547721 |
| 4849 | CNOT3 | CCR4-NOT transcription complex subunit 3 | -1.748972151 | 0.024701992 |
| 10330 | CNPY2 | canopy FGF signaling regulator 2 | 0.960098914 | 0.035042707 |
| 170712 | COX7B2 | cytochrome c oxidase subunit 7B2 | 1.575851683 | 0.040615282 |
| 1351 | COX8A | cytochrome c oxidase subunit 8A | 1.002522644 | 0.099202608 |
| 1356 | CP | ceruloplasmin (ferroxidase) | 1.6634758 | 0.040483698 |
| 132864 | CPEB2 | cytoplasmic polyadenylation element binding protein 2 | -1.038758547 | 0.040483698 |
| 1368 | CPM | carboxypeptidase M | 1.873834941 | 0.076600743 |
| 1508 | CTSB | cathepsin B | 1.227827647 | 0.034999155 |
| 1510 | CTSE | cathepsin E | 1.145497457 | 0.023791032 |
| 3627 | CXCL10 | C-X-C motif chemokine ligand 10 | 1.944758738 | 0.02122451 |
| 3576 | CXCL8 | C-X-C motif chemokine ligand 8 | 1.721923904 | 0.033252629 |
| 55086 | CXorf57 | chromosome X open reading frame 57 | -0.961174844 | 0.091091149 |
| 1535 | CYBA | cytochrome b-245, alpha polypeptide | 1.026378684 | 0.011701916 |
| 1591 | CYP24A1 | cytochrome P450 family 24 subfamily A member 1 | 1.416897676 | 0.024362931 |
| 57834 | CYP4F11 | cytochrome P450 family 4 subfamily F member 11 | 2.217207803 | 0.01537599 |
| 3491 | CYR61 | cysteine rich angiogenic inducer 61 | -1.140038603 | 0.008903491 |
| 166614 | DCLK2 | doublecortin like kinase 2 | -1.063291004 | 0.044247978 |
| 55601 | DDX60 | DEXD/H-box helicase 60 | 1.387220675 | 0.040483698 |
| 57706 | DENND1A | DENN domain containing 1A | -0.954503395 | 0.009407116 |
| 51009 | DERL2 | derlin 2 | 0.917645335 | 0.079278572 |
| 10170 | DHRS9 | dehydrogenase/reductase (SDR family) member 9 | 1.453920844 | 0.02693268 |
| 2039 | DMTN | dematin actin binding protein | -1.345441003 | 0.009814848 |
| 5611 | DNAJC3 | DnaJ heat shock protein family (Hsp40) member C3 | 1.021776089 | 0.052579421 |
| 22826 | DNAJC8 | DnaJ heat shock protein family (Hsp40) member C8 | 1.01488079 | 0.045320964 |
| 80005 | DOCK5 | dedicator of cytokinesis 5 | 1.326520011 | 0.090875484 |
| 1848 | DUSP6 | dual specificity phosphatase 6 | 1.018420236 | 0.035616373 |
| 9149 | DYRK1B | dual specificity tyrosine phosphorylation regulated kinase 1B | -1.201025442 | 0.046207018 |
| 10682 | EBP | emopamil binding protein (sterol isomerase) | 0.98395512 | 0.087142461 |
| 1891 | ECH1 | enoyl-CoA hydratase 1, peroxisomal | -2.596664088 | 0.05167349 |
| 1909 | EDNRA | endothelin receptor type A | 1.448879348 | 0.049610609 |
| 163126 | EID2 | EP300 interacting inhibitor of differentiation 2 | -1.427662349 | 0.096243151 |
| 2012 | EMP1 | epithelial membrane protein 1 | 1.633200818 | 0.018936374 |
| 2034 | EPAS1 | endothelial PAS domain protein 1 | 1.749751013 | 0.01382131 |
| 80314 | EPC1 | enhancer of polycomb homolog 1 | -0.912706249 | 0.02380447 |
| 255324 | EPGN | epithelial mitogen | -2.126906911 | 0.07912276 |
| 51575 | ESF1 | ESF1 nucleolar pre-rRNA processing protein homolog | 1.002593098 | 0.019008949 |
| 2139 | EYA2 | EYA transcriptional coactivator and phosphatase 2 | 1.018523444 | 0.064721574 |
| 2151 | F2RL2 | coagulation factor II thrombin receptor like 2 | 1.889027991 | 0.013331274 |
| 54491 | FAM105A | family with sequence similarity 105 member A | 1.653590307 | 0.023396946 |
| 116496 | FAM129A | family with sequence similarity 129 member A | -1.696113151 | 0.007405838 |
| 2257 | FGF12 | fibroblast growth factor 12 | -1.710255319 | 0.029915586 |
| 2286 | FKBP2 | FK506 binding protein 2 | 1.084946885 | 0.038252089 |
| 2335 | FN1 | fibronectin 1 | -1.911730784 | 0.034422082 |
| 23048 | FNBP1 | formin binding protein 1 | -1.199486687 | 0.01987844 |
| 2348 | FOLR1 | folate receptor 1 (adult) | 2.385763457 | 0.067013055 |
| 2296 | FOXC1 | forkhead box C1 | -1.42553534 | 0.015760839 |
| 80144 | FRAS1 | Fraser extracellular matrix complex subunit 1 | -2.049775162 | 0.024078668 |
| 84978 | FRMD5 | FERM domain containing 5 | -0.948957917 | 0.03021399 |
| 9615 | GDA | guanine deaminase | -1.222998292 | 0.019008949 |
| 2674 | GFRA1 | GDNF family receptor alpha 1 | -1.752551003 | 0.010319288 |
| 2706 | GJB2 | gap junction protein beta 2 | 2.281220767 | 0.047022055 |
| 2717 | GLA | galactosidase alpha | 0.943004033 | 0.099715992 |
| 2736 | GLI2 | GLI family zinc finger 2 | -0.927857672 | 0.032515392 |
| 84803 | GPAT3 | glycerol-3-phosphate acyltransferase 3 | 1.090840801 | 0.079692386 |
| 9052 | GPRC5A | G protein-coupled receptor class C group 5 member A | 1.042798186 | 0.099202608 |
| 2876 | GPX1 | glutathione peroxidase 1 | -1.087438059 | 0.040594086 |
| 2931 | GSK3A | glycogen synthase kinase 3 alpha | -1.126052927 | 0.021553078 |
| 27198 | HCAR1 | hydroxycarboxylic acid receptor 1 | 1.378553954 | 0.009814848 |
| 3081 | HGD | homogentisate 1,2-dioxygenase | 1.426922011 | 0.060122498 |
| 8335 | HIST1H2AB | histone cluster 1, H2ab | 1.393698306 | 0.031055687 |
| 85235 | HIST1H2AH | histone cluster 1, H2ah | 1.130689675 | 0.076079226 |
| 8342 | HIST1H2BM | histone cluster 1, H2bm | 1.277818507 | 0.044247978 |
| 8350 | HIST1H3A | histone cluster 1, H3a | 1.091726719 | 0.055593841 |
| 8968 | HIST1H3F | histone cluster 1, H3f | 1.429632374 | 0.029593936 |
| 8354 | HIST1H3I | histone cluster 1, H3i | -3.035312911 | 0.045491281 |
| 9324 | HMGN3 | high mobility group nucleosomal binding domain 3 | 0.940004614 | 0.098834032 |
| 3161 | HMMR | hyaluronan mediated motility receptor | 1.189053556 | 0.024663673 |
| 3212 | HOXB2 | homeobox B2 | -1.465834649 | 0.042351159 |
| 51170 | HSD17B11 | hydroxysteroid (17-beta) dehydrogenase 11 | 1.103561701 | 0.082687849 |
| 51171 | HSD17B14 | hydroxysteroid (17-beta) dehydrogenase 14 | 1.139400357 | 0.048871455 |
| 3306 | HSPA2 | heat shock protein family A (Hsp70) member 2 | 1.126127744 | 0.008903491 |
| 3383 | ICAM1 | intercellular adhesion molecule 1 | 0.938338979 | 0.072377194 |
| 3429 | IFI27 | interferon, alpha-inducible protein 27 | 1.787802598 | 0.009407116 |
| 10964 | IFI44L | interferon induced protein 44 like | 1.701138744 | 0.026934047 |
| 2537 | IFI6 | interferon, alpha-inducible protein 6 | 3.172517762 | 0.01537599 |
| 3437 | IFIT3 | interferon induced protein with tetratricopeptide repeats 3 | 1.55552482 | 0.098838561 |
| 8519 | IFITM1 | interferon induced transmembrane protein 1 | 2.376380873 | 0.0075272 |
| 10410 | IFITM3 | interferon induced transmembrane protein 3 | 1.639556375 | 0.032515392 |
| 340198 | IFITM4P | interferon induced transmembrane protein 4 pseudogene | 1.898746911 | 0.057515754 |
| 3485 | IGFBP2 | insulin like growth factor binding protein 2 | 0.969471021 | 0.042351159 |
| 3488 | IGFBP5 | insulin like growth factor binding protein 5 | -0.995786195 | 0.031055687 |
| 3606 | IL18 | interleukin 18 | 1.506392939 | 0.070308769 |
| 7850 | IL1R2 | interleukin 1 receptor type 2 | 1.383007083 | 0.060854089 |
| 3394 | IRF8 | interferon regulatory factor 8 | 1.444052674 | 0.056090043 |
| 9636 | ISG15 | ISG15 ubiquitin-like modifier | 1.204785454 | 0.055959924 |
| 3673 | ITGA2 | integrin subunit alpha 2 | -1.123534163 | 0.074699808 |
| 3690 | ITGB3 | integrin subunit beta 3 | 1.946590597 | 0.052579421 |
| 133746 | JMY | junction mediating and regulatory protein, p53 cofactor | -1.08729643 | 0.010821422 |
| 3786 | KCNQ3 | potassium voltage-gated channel subfamily Q member 3 | -2.43264513 | 0.009407116 |
| 3787 | KCNS1 | potassium voltage-gated channel modifier subfamily S member 1 | 1.928526867 | 0.016770484 |
| 23135 | KDM6B | lysine demethylase 6B | -0.932836567 | 0.079692386 |
| 9710 | KIAA0355 | KIAA0355 | -1.1781172 | 0.046499198 |
| 25758 | KIAA1549L | KIAA1549-like | -2.13353643 | 0.02630864 |
| 158405 | KIAA1958 | KIAA1958 | -1.18773972 | 0.01987844 |
| 547 | KIF1A | kinesin family member 1A | -0.954843764 | 0.035042707 |
| 9585 | KIF20B | kinesin family member 20B | 1.328606247 | 0.025165898 |
| 114818 | KLHL29 | kelch like family member 29 | -0.991621417 | 0.018335946 |
| 9757 | KMT2B | lysine methyltransferase 2B | -0.943981372 | 0.051309396 |
| 3909 | LAMA3 | laminin subunit alpha 3 | 1.519049018 | 0.007405838 |
| 27074 | LAMP3 | lysosomal associated membrane protein 3 | -1.209926788 | 0.076661305 |
| 389421 | LIN28B | lin-28 homolog B | -1.84112296 | 0.03339405 |
| 285548 | LINC01096 | long intergenic non-protein coding RNA 1096 | -0.916178274 | 0.075443116 |
| 100505633 | LINC01133 | long intergenic non-protein coding RNA 1133 | -1.074359033 | 0.090668054 |
| 101929623 | LINC01215 | long intergenic non-protein coding RNA 1215 | 1.133963009 | 0.017612071 |
| 101928689 | LINC01424 | long intergenic non-protein coding RNA 1424 | 0.90580426 | 0.050136508 |
| 100129995 | LINC01460 | long intergenic non-protein coding RNA 1460 | 0.942709852 | 0.090668054 |
| 3996 | LLGL1 | lethal giant larvae homolog 1, scribble cell polarity complex component | -0.942134513 | 0.092841833 |
| 100129233 | LOC100129233 | uncharacterized LOC100129233 | 1.33560667 | 0.026014755 |
| 100652999 | LOC100652999 | uncharacterized LOC100652999 | -1.035145286 | 0.057549239 |
| 101927418 | LOC101927418 | uncharacterized LOC101927418 | 2.05121254 | 0.087184406 |
| 101928045 | LOC101928045 | uncharacterized LOC101928045 | 1.274612128 | 0.046714797 |
| 101928277 | LOC101928277 | uncharacterized LOC101928277 | 1.068278314 | 0.079762966 |
| 101928694 | LOC101928694 | uncharacterized LOC101928694 | 0.981768012 | 0.052010946 |
| 102724885 | LOC102724885 | uncharacterized LOC102724885 | 1.407315179 | 0.055959924 |
| 399900 | LOC399900 | uncharacterized LOC399900 | 0.940590828 | 0.044423075 |
| 645513 | LOC645513 | uncharacterized LOC645513 | -0.919839632 | 0.021583233 |
| 731157 | LOC731157 | uncharacterized LOC731157 | 1.337614162 | 0.024078668 |
| 164832 | LONRF2 | LON peptidase N-terminal domain and ring finger 2 | -1.721358394 | 0.008903491 |
| 4017 | LOXL2 | lysyl oxidase like 2 | 1.109553741 | 0.076661305 |
| 54947 | LPCAT2 | lysophosphatidylcholine acyltransferase 2 | 1.938858283 | 0.078744194 |
| 79414 | LRFN3 | leucine rich repeat and fibronectin type III domain containing 3 | -0.901609088 | 0.072377194 |
| 286343 | LURAP1L | leucine rich adaptor protein 1-like | -1.684101574 | 0.002656143 |
| 4061 | LY6E | lymphocyte antigen 6 complex, locus E | 1.041401508 | 0.061268862 |
| 116372 | LYPD1 | LY6/PLAUR domain containing 1 | -1.628330247 | 0.009911004 |
| 126868 | MAB21L3 | mab-21-like 3 (C. elegans) | 1.716911163 | 0.009911004 |
| 10916 | MAGED2 | MAGE family member D2 | 0.951537654 | 0.024663673 |
| 7873 | MANF | mesencephalic astrocyte derived neurotrophic factor | 0.907817042 | 0.053971228 |
| 4131 | MAP1B | microtubule associated protein 1B | -1.250552068 | 0.021127043 |
| 643246 | MAP1LC3B2 | microtubule associated protein 1 light chain 3 beta 2 | -1.259460971 | 0.099786791 |
| 4294 | MAP3K10 | mitogen-activated protein kinase kinase kinase 10 | -1.353315656 | 0.044195147 |
| 83742 | MARVELD1 | MARVEL domain containing 1 | 0.967561713 | 0.07912276 |
| 79772 | MCTP1 | multiple C2 and transmembrane domain containing 1 | 1.420394943 | 0.063185718 |
| 2122 | MECOM | MDS1 and EVI1 complex locus | 1.457482396 | 0.03864656 |
| 25840 | METTL7A | methyltransferase like 7A | 1.007632141 | 0.071753066 |
| 84879 | MFSD2A | major facilitator superfamily domain containing 2A | 1.136852487 | 0.012148333 |
| 146664 | MGAT5B | mannosyl (alpha-1,6-)-glycoprotein beta-1,6-N-acetyl-glucosaminyltransferase, isozyme B | -0.973888258 | 0.024078668 |
| 4258 | MGST2 | microsomal glutathione S-transferase 2 | 1.301989878 | 0.021823749 |
| 406949 | MIR15B | microRNA 15b | 1.329001875 | 0.057395772 |
| 554202 | MIR31HG | MIR31 host gene | -1.3464007 | 0.040204907 |
| 100422909 | MIR4295 | microRNA 4295 | 1.282907986 | 0.040212444 |
| 100616160 | MIR4655 | microRNA 4655 | -1.105015328 | 0.064721574 |
| 100313771 | MIR548F2 | microRNA 548f-2 | 1.480928765 | 0.043669517 |
| 100302277 | MIR548I2 | microRNA 548i-2 | -1.096709171 | 0.078744194 |
| 100313770 | MIR548K | microRNA 548k | 1.772961429 | 0.0075272 |
| 693229 | MIR644A | microRNA 644a | 1.4218166 | 0.050136508 |
| 407046 | MIR9-1 | microRNA 9-1 | 1.269139096 | 0.090802996 |
| 283078 | MKX | mohawk homeobox | -1.613117046 | 0.00619556 |
| 55329 | MNS1 | meiosis specific nuclear structural 1 | 1.113075542 | 0.030724287 |
| 79710 | MORC4 | MORC family CW-type zinc finger 4 | -1.242990248 | 0.029331438 |
| 29093 | MRPL22 | mitochondrial ribosomal protein L22 | 0.984497766 | 0.067265967 |
| 92154 | MTSS1L | metastasis suppressor 1-like | -0.961269961 | 0.029123846 |
| 222166 | MTURN | maturin, neural progenitor differentiation regulator homolog (Xenopus) | -1.036784673 | 0.076600743 |
| 4599 | MX1 | MX dynamin like GTPase 1 | 1.473078627 | 0.048203172 |
| 25878 | MXRA5 | matrix-remodelling associated 5 | -1.840254921 | 0.017717492 |
| 4668 | NAGA | N-acetylgalactosaminidase, alpha- | 0.925108875 | 0.040483698 |
| 4700 | NDUFA6 | NADH:ubiquinone oxidoreductase subunit A6 | 1.104531425 | 0.057395772 |
| 4773 | NFATC2 | nuclear factor of activated T-cells, cytoplasmic, calcineurin-dependent 2 | -1.010912643 | 0.025560257 |
| 22795 | NID2 | nidogen 2 | 1.38079317 | 0.026014755 |
| 50507 | NOX4 | NADPH oxidase 4 | 2.110292035 | 0.050136508 |
| 64067 | NPAS3 | neuronal PAS domain protein 3 | -1.496200862 | 0.012148333 |
| 4907 | NT5E | 5'-nucleotidase ecto | -1.826533903 | 0.002881538 |
| 389493 | NUPR2 | nuclear protein 2, transcriptional regulator | -1.757173194 | 0.025392257 |
| 4938 | OAS1 | 2'-5'-oligoadenylate synthetase 1 | 1.073068971 | 0.039089657 |
| 4983 | OPHN1 | oligophrenin 1 | -1.140973624 | 0.01537599 |
| 58503 | OPRPN | opiorphin prepropeptide | -2.646088084 | 0.010427045 |
| 26247 | OR2L1P | olfactory receptor family 2 subfamily L member 1 pseudogene | -0.950937024 | 0.082391534 |
| 29095 | ORMDL2 | ORMDL sphingolipid biosynthesis regulator 2 | 1.241239109 | 0.040204907 |
| 51633 | OTUD6B | OTU domain containing 6B | 1.105308264 | 0.060579497 |
| 5031 | P2RY6 | pyrimidinergic receptor P2Y6 | 1.178618343 | 0.024078668 |
| 5033 | P4HA1 | prolyl 4-hydroxylase subunit alpha 1 | 0.965815704 | 0.014511128 |
| 23241 | PACS2 | phosphofurin acidic cluster sorting protein 2 | -0.965384792 | 0.040533952 |
| 90737 | PAGE5 | PAGE family member 5 | 1.020192483 | 0.065214823 |
| 117583 | PARD3B | par-3 family cell polarity regulator beta | -0.91242864 | 0.068376652 |
| 55872 | PBK | PDZ binding kinase | 0.946948856 | 0.079692386 |
| 5087 | PBX1 | pre-B-cell leukemia homeobox 1 | 1.230769437 | 0.069970566 |
| 57526 | PCDH19 | protocadherin 19 | -1.415451732 | 0.07149909 |
| 8654 | PDE5A | phosphodiesterase 5A | -1.212622875 | 0.022743829 |
| 80055 | PGAP1 | post-GPI attachment to proteins 1 | -1.09635066 | 0.015760839 |
| 5253 | PHF2 | PHD finger protein 2 | -1.233763544 | 0.044195147 |
| 5266 | PI3 | peptidase inhibitor 3 | 5.995653443 | 0.050136508 |
| 5569 | PKIA | protein kinase (cAMP-dependent, catalytic) inhibitor alpha | -0.962293041 | 0.064721574 |
| 23129 | PLXND1 | plexin D1 | 1.013983387 | 0.026014755 |
| 23126 | POGZ | pogo transposable element with ZNF domain | -0.933008219 | 0.029331438 |
| 445582 | POTEE | POTE ankyrin domain family member E | 1.155651361 | 0.045102723 |
| 728378 | POTEF | POTE ankyrin domain family member F | 1.186881976 | 0.057395772 |
| 5563 | PRKAA2 | protein kinase AMP-activated catalytic subunit alpha 2 | -1.197341703 | 0.065052444 |
| 10544 | PROCR | protein C receptor | 1.626512106 | 0.050136508 |
| 148137 | PROSER3 | proline and serine rich 3 | -0.923778763 | 0.085376991 |
| 80164 | PRR36 | proline rich 36 | -1.361587936 | 0.009882448 |
| 7916 | PRRC2A | proline rich coiled-coil 2A | -1.064247417 | 0.037300229 |
| 5784 | PTPN14 | protein tyrosine phosphatase, non-receptor type 14 | -1.012044301 | 0.026014755 |
| 5806 | PTX3 | pentraxin 3 | 2.909403497 | 0.019367865 |
| 29108 | PYCARD | PYD and CARD domain containing | 1.355425544 | 0.0580646 |
| 79912 | PYROXD1 | pyridine nucleotide-disulphide oxidoreductase domain 1 | 1.117543834 | 0.039089657 |
| 5892 | RAD51D | RAD51 paralog D | 1.674716999 | 0.061885205 |
| 101926888 | RALY-AS1 | RALY antisense RNA 1 | 0.912905811 | 0.072640808 |
| 2889 | RAPGEF1 | Rap guanine nucleotide exchange factor 1 | -1.099734395 | 0.014511128 |
| 64864 | RFX7 | regulatory factor X7 | -0.959734295 | 0.013208959 |
| 22999 | RIMS1 | regulating synaptic membrane exocytosis 1 | -1.358893779 | 0.050362597 |
| 255488 | RNF144B | ring finger protein 144B | -0.930119798 | 0.060122498 |
| 51255 | RNF181 | ring finger protein 181 | 1.020898155 | 0.036270526 |
| 22838 | RNF44 | ring finger protein 44 | -1.11739492 | 0.009814848 |
| 26824 | RNU11 | RNA, U11 small nuclear | 2.153208391 | 0.02324191 |
| 100151683 | RNU4ATAC | RNA, U4atac small nuclear (U12-dependent splicing) | 1.451132654 | 0.01408342 |
| 26830 | RNU5D-1 | RNA, U5D small nuclear 1 | 1.870891895 | 0.026465033 |
| 26828 | RNU5F-1 | RNA, U5F small nuclear 1 | 2.369653522 | 0.043669517 |
| 100873774 | RNU6-71P | RNA, U6 small nuclear 71, pseudogene | 0.971461631 | 0.064721574 |
| 100151684 | RNU6ATAC | RNA, U6atac small nuclear (U12-dependent splicing) | 1.19839618 | 0.013197002 |
| 101954266 | RNVU1-14 | RNA, variant U1 small nuclear 14 | 2.264264271 | 0.008575124 |
| 101954267 | RNVU1-15 | RNA, variant U1 small nuclear 15 | 0.934915193 | 0.079692386 |
| 101954277 | RNVU1-19 | RNA, variant U1 small nuclear 19 | 1.44760175 | 0.021583233 |
| 101954264 | RNVU1-4 | RNA, variant U1 small nuclear 4 | 0.965196124 | 0.024701992 |
| 6120 | RPE | ribulose-5-phosphate-3-epimerase | 0.990063093 | 0.0799589 |
| 6171 | RPL41 | ribosomal protein L41 | 1.041663713 | 0.042351159 |
| 100506007 | RTCA-AS1 | RTCA antisense RNA 1 | 0.995780332 | 0.072038558 |
| 55095 | SAMD4B | sterile alpha motif domain containing 4B | -1.786519375 | 0.068627046 |
| 25939 | SAMHD1 | SAM domain and HD domain 1 | 0.927925547 | 0.065214823 |
| 692148 | SCARNA10 | small Cajal body-specific RNA 10 | 1.691381638 | 0.009814848 |
| 692149 | SCARNA14 | small Cajal body-specific RNA 14 | 1.140066846 | 0.038252089 |
| 677769 | SCARNA17 | small Cajal body-specific RNA 17 | 1.18848619 | 0.009882448 |
| 677770 | SCARNA22 | small Cajal body-specific RNA 22 | 1.5303912 | 0.01537599 |
| 677771 | SCARNA4 | small Cajal body-specific RNA 4 | 1.198538087 | 0.009814848 |
| 677775 | SCARNA5 | small Cajal body-specific RNA 5 | 0.956007461 | 0.034999155 |
| 677772 | SCARNA6 | small Cajal body-specific RNA 6 | 1.348275486 | 0.031720021 |
| 677776 | SCARNA8 | small Cajal body-specific RNA 8 | 1.9167935 | 0.011701916 |
| 100158262 | SCARNA9L | small Cajal body-specific RNA 9-like | 1.268608171 | 0.007405838 |
| 23753 | SDF2L1 | stromal cell derived factor 2 like 1 | 1.062772222 | 0.079278572 |
| 8991 | SELENBP1 | selenium binding protein 1 | 1.203461206 | 0.009814848 |
| 10512 | SEMA3C | semaphorin 3C | -1.112992604 | 0.01382131 |
| 118980 | SFXN2 | sideroflexin 2 | 0.973103295 | 0.048067187 |
| 23094 | SIPA1L3 | signal-induced proliferation-associated 1 like 3 | -1.72931006 | 0.069439173 |
| 51804 | SIX4 | SIX homeobox 4 | -0.965209963 | 0.032175028 |
| 151473 | SLC16A14 | solute carrier family 16 member 14 | -1.448969554 | 0.099430199 |
| 6507 | SLC1A3 | solute carrier family 1 member 3 | 2.40019415 | 0.026014755 |
| 6574 | SLC20A1 | solute carrier family 20 member 1 | 1.301391298 | 0.078778059 |
| 79085 | SLC25A23 | solute carrier family 25 member 23 | -0.995305824 | 0.052579421 |
| 81894 | SLC25A28 | solute carrier family 25 member 28 | 1.054492717 | 0.063185718 |
| 1836 | SLC26A2 | solute carrier family 26 member 2 | 1.577635299 | 0.095404762 |
| 6513 | SLC2A1 | solute carrier family 2 member 1 | 1.083285086 | 0.007405838 |
| 55974 | SLC50A1 | solute carrier family 50 member 1 | 0.994980488 | 0.064721574 |
| 81796 | SLCO5A1 | solute carrier organic anion transporter family member 5A1 | -1.442385303 | 0.008903491 |
| 6590 | SLPI | secretory leukocyte peptidase inhibitor | 2.151642617 | 0.029331438 |
| 64750 | SMURF2 | SMAD specific E3 ubiquitin protein ligase 2 | -1.030606805 | 0.010319288 |
| 100170227 | SNAR-D | small ILF3/NF90-associated RNA D | 0.997059229 | 0.031940094 |
| 100507303 | SNHG19 | small nucleolar RNA host gene 19 | 1.235088641 | 0.034149818 |
| 677800 | SNORA12 | small nucleolar RNA, H/ACA box 12 | 1.104952652 | 0.007405838 |
| 654322 | SNORA13 | small nucleolar RNA, H/ACA box 13 | 1.489429478 | 0.026528173 |
| 677802 | SNORA14B | small nucleolar RNA, H/ACA box 14B | 0.915370777 | 0.034241124 |
| 677794 | SNORA2B | small nucleolar RNA, H/ACA box 2B | 1.392052653 | 0.072080146 |
| 594839 | SNORA33 | small nucleolar RNA, H/ACA box 33 | 1.998872655 | 0.017612071 |
| 677820 | SNORA38 | small nucleolar RNA, H/ACA box 38 | 1.161537625 | 0.069439173 |
| 100124536 | SNORA38B | small nucleolar RNA, H/ACA box 38B | 2.006514766 | 0.050136508 |
| 677826 | SNORA3B | small nucleolar RNA, H/ACA box 3B | 1.161590074 | 0.050242133 |
| 677827 | SNORA46 | small nucleolar RNA, H/ACA box 46 | 1.85936191 | 0.017726252 |
| 677828 | SNORA47 | small nucleolar RNA, H/ACA box 47 | 0.915979332 | 0.089487005 |
| 677830 | SNORA50A | small nucleolar RNA, H/ACA box 50A | 0.927164373 | 0.019928657 |
| 677842 | SNORA50C | small nucleolar RNA, H/ACA box 50C | 1.112892156 | 0.048854869 |
| 677834 | SNORA55 | small nucleolar RNA, H/ACA box 55 | 0.998332687 | 0.057500471 |
| 677837 | SNORA60 | small nucleolar RNA, H/ACA box 60 | 1.923838201 | 0.074199983 |
| 26783 | SNORA65 | small nucleolar RNA, H/ACA box 65 | 1.060313075 | 0.060634869 |
| 100379132 | SNORA70G | small nucleolar RNA, H/ACA box 70G | 1.282600149 | 0.01537599 |
| 677840 | SNORA71D | small nucleolar RNA, H/ACA box 71D | 1.231577426 | 0.090802996 |
| 677823 | SNORA80E | small nucleolar RNA, H/ACA box 80E | 1.799543889 | 0.081374661 |
| 692229 | SNORD105 | small nucleolar RNA, C/D box 105 | 0.972086944 | 0.01537599 |
| 692214 | SNORD111 | small nucleolar RNA, C/D box 111 | 1.684544107 | 0.01537599 |
| 692233 | SNORD117 | small nucleolar RNA, C/D box 117 | 1.305285409 | 0.029593936 |
| 692057 | SNORD12 | small nucleolar RNA, C/D box 12 | 2.260640587 | 0.011701916 |
| 100113384 | SNORD123 | small nucleolar RNA, C/D box 123 | 1.791730069 | 0.032515392 |
| 100113393 | SNORD12B | small nucleolar RNA, C/D box 12B | 2.234517932 | 0.007405838 |
| 26765 | SNORD12C | small nucleolar RNA, C/D box 12C | 2.5081788 | 0.009814848 |
| 26822 | SNORD14A | small nucleolar RNA, C/D box 14A | 1.296247261 | 0.009407116 |
| 692086 | SNORD17 | small nucleolar RNA, C/D box 17 | 1.365813548 | 0.01987844 |
| 677849 | SNORD1B | small nucleolar RNA, C/D box 1B | 1.249805476 | 0.091808393 |
| 26812 | SNORD37 | small nucleolar RNA, C/D box 37 | 0.936242578 | 0.045024999 |
| 780853 | SNORD3C | small nucleolar RNA, C/D box 3C | 2.020562746 | 0.002656143 |
| 26810 | SNORD41 | small nucleolar RNA, C/D box 41 | 1.035959003 | 0.034149818 |
| 26798 | SNORD51 | small nucleolar RNA, C/D box 51 | 1.834935444 | 0.032175028 |
| 692107 | SNORD66 | small nucleolar RNA, C/D box 66 | 1.20274595 | 0.057549239 |
| 692111 | SNORD71 | small nucleolar RNA, C/D box 71 | 1.119021101 | 0.026934047 |
| 619564 | SNORD72 | small nucleolar RNA, C/D box 72 | 1.565164656 | 0.039089657 |
| 319103 | SNORD8 | small nucleolar RNA, C/D box 8 | 1.079959924 | 0.044247978 |
| 116937 | SNORD83A | small nucleolar RNA, C/D box 83A | 1.187899024 | 0.039089657 |
| 692207 | SNORD91A | small nucleolar RNA, C/D box 91A | 2.251462877 | 0.044247978 |
| 6628 | SNRPB | small nuclear ribonucleoprotein polypeptides B and B1 | 0.924429016 | 0.043669517 |
| 30837 | SOCS7 | suppressor of cytokine signaling 7 | -1.008371241 | 0.021285016 |
| 345079 | SOWAHB | sosondowah ankyrin repeat domain family member B | -0.931266627 | 0.031959091 |
| 6722 | SRF | serum response factor | -0.970283439 | 0.01537599 |
| 6717 | SRI | sorcin | 1.785006208 | 0.045491281 |
| 6742 | SSBP1 | single-stranded DNA binding protein 1, mitochondrial | 0.915223602 | 0.088079447 |
| 10610 | ST6GALNAC2 | ST6 (alpha-N-acetyl-neuraminyl-2,3-beta-galactosyl-1,3)-N-acetylgalactosaminide alpha-2,6-sialyltransferase 2 | -1.236346865 | 0.077541136 |
| 6769 | STAC | SH3 and cysteine rich domain | -0.995077702 | 0.061268862 |
| 6781 | STC1 | stanniocalcin 1 | 2.897931431 | 0.057500471 |
| 79689 | STEAP4 | STEAP4 metalloreductase | 1.450788931 | 0.015760839 |
| 56241 | SUSD2 | sushi domain containing 2 | 1.647990415 | 0.05167349 |
| 10388 | SYCP2 | synaptonemal complex protein 2 | 1.996803234 | 0.064721574 |
| 50840 | TAS2R14 | taste 2 receptor member 14 | 1.126807914 | 0.01537599 |
| 51186 | TCEAL9 | transcription elongation factor A like 9 | 1.116521892 | 0.055276931 |
| 6929 | TCF3 | transcription factor 3 | -0.971111229 | 0.02693268 |
| 83439 | TCF7L1 | transcription factor 7 like 1 | -0.935506205 | 0.039089657 |
| 163589 | TDRD5 | tudor domain containing 5 | -0.907703111 | 0.086092391 |
| 56154 | TEX15 | testis expressed 15 | 1.271487022 | 0.035042707 |
| 7052 | TGM2 | transglutaminase 2 | 2.465937824 | 6.31689E-04 |
| 9333 | TGM5 | transglutaminase 5 | 1.898932375 | 0.033624233 |
| 200765 | TIGD1 | tigger transposable element derived 1 | 0.973087166 | 0.014278678 |
| 92609 | TIMM50 | translocase of inner mitochondrial membrane 50 | -1.162796717 | 0.083806849 |
| 7082 | TJP1 | tight junction protein 1 | -1.003819675 | 0.022617082 |
| 7083 | TK1 | thymidine kinase 1, soluble | 1.298228379 | 0.02693268 |
| 7084 | TK2 | thymidine kinase 2, mitochondrial | 1.082073158 | 0.017547721 |
| 7098 | TLR3 | toll like receptor 3 | 1.143733132 | 0.009072033 |
| 100507421 | TMEM178B | transmembrane protein 178B | -1.167951381 | 0.009407116 |
| 29100 | TMEM208 | transmembrane protein 208 | 1.365484308 | 0.034999155 |
| 83604 | TMEM47 | transmembrane protein 47 | -1.936001341 | 0.002656143 |
| 23585 | TMEM50A | transmembrane protein 50A | 1.019928075 | 0.064721574 |
| 29767 | TMOD2 | tropomodulin 2 | -1.063420664 | 0.042288797 |
| 5651 | TMPRSS15 | transmembrane protease, serine 15 | -4.929733417 | 0.052586372 |
| 56649 | TMPRSS4 | transmembrane protease, serine 4 | 1.395788977 | 0.021941871 |
| 222699 | TOB2P1 | transducer of ERBB2, 2 pseudogene 1 | -1.012508125 | 0.042351159 |
| 9760 | TOX | thymocyte selection associated high mobility group box | -1.271675277 | 0.052586372 |
| 27324 | TOX3 | TOX high mobility group box family member 3 | -1.015650639 | 0.045701067 |
| 27010 | TPK1 | thiamin pyrophosphokinase 1 | -1.09865842 | 0.034999155 |
| 283392 | TRHDE-AS1 | TRHDE antisense RNA 1 | 0.900348074 | 0.021349686 |
| 10103 | TSPAN1 | tetraspanin 1 | 2.202893265 | 0.043344501 |
| 10382 | TUBB4A | tubulin beta 4A class IVa | -1.571158738 | 0.097331402 |
| 10190 | TXNDC9 | thioredoxin domain containing 9 | 1.041905555 | 0.069970566 |
| 55075 | UACA | uveal autoantigen with coiled-coil domains and ankyrin repeats | 0.939399868 | 0.012148333 |
| 54887 | UHRF1BP1 | UHRF1 binding protein 1 | -1.02840055 | 0.017101094 |
| 389856 | USP27X | ubiquitin specific peptidase 27, X-linked | -0.990969567 | 0.083211481 |
| 8673 | VAMP8 | vesicle associated membrane protein 8 | 0.919090002 | 0.032515392 |
| 25806 | VAX2 | ventral anterior homeobox 2 | -0.933012752 | 0.078935496 |
| 5212 | VIT | vitrin | -1.864312752 | 0.043741224 |
| 56664 | VTRNA1-1 | vault RNA 1-1 | 1.586880883 | 0.050136508 |
| 56663 | VTRNA1-2 | vault RNA 1-2 | 2.995112811 | 0.011701916 |
| 56662 | VTRNA1-3 | vault RNA 1-3 | 1.717267565 | 0.017547721 |
| 100126299 | VTRNA2-1 | vault RNA 2-1 | 2.412714393 | 0.01408342 |
| 8936 | WASF1 | WAS protein family member 1 | -1.424154194 | 0.009072033 |
| 10406 | WFDC2 | WAP four-disulfide core domain 2 | 1.312492808 | 0.040204907 |
| 8839 | WISP2 | WNT1 inducible signaling pathway protein 2 | 1.070527787 | 0.026946365 |
| 65268 | WNK2 | WNK lysine deficient protein kinase 2 | -1.150425644 | 0.090802996 |
| 9502 | XAGE2 | X antigen family member 2 | 1.885268043 | 0.012148333 |
| 7498 | XDH | xanthine dehydrogenase | 2.298853621 | 0.019442395 |
| 57510 | XPO5 | exportin 5 | -0.921709529 | 0.01537599 |
| 57002 | YAE1D1 | Yae1 domain containing 1 | 0.900806654 | 0.062310513 |
| 54877 | ZCCHC2 | zinc finger CCHC-type containing 2 | -0.973510865 | 0.009882448 |
| 441951 | ZFAS1 | ZNFX1 antisense RNA 1 | 1.15200266 | 0.048067187 |
| 677 | ZFP36L1 | ZFP36 ring finger protein-like 1 | -1.003155404 | 0.085723138 |
| 100873922 | ZFX-AS1 | ZFX antisense RNA 1 | 0.98739656 | 0.033252629 |
| 91975 | ZNF300 | zinc finger protein 300 | 1.049865409 | 0.084506416 |
| 55422 | ZNF331 | zinc finger protein 331 | -1.028048857 | 0.033252629 |
| 55786 | ZNF415 | zinc finger protein 415 | -0.901685084 | 0.090875484 |
| 57473 | ZNF512B | zinc finger protein 512B | -1.026879455 | 0.032220305 |
| 84503 | ZNF527 | zinc finger protein 527 | -1.062875888 | 0.043669517 |
| 148268 | ZNF570 | zinc finger protein 570 | -1.711856584 | 0.009312563 |
| 199704 | ZNF585A | zinc finger protein 585A | -1.208400787 | 0.023396946 |
| 84622 | ZNF594 | zinc finger protein 594 | -0.922111063 | 0.089487005 |
| 7552 | ZNF711 | zinc finger protein 711 | -1.828390803 | 0.067855301 |
| 643955 | ZNF733P | zinc finger protein 733, pseudogene | 1.044873442 | 0.038139938 |
| 645700 | ZNF890P | zinc finger protein 890, pseudogene | -1.007171143 | 0.058836744 |
| 81931 | ZNF93 | zinc finger protein 93 | -1.059242085 | 0.043669517 |
