# Supplementary material for: The epigenetic factor BORIS (CTCFL) controls the androgen receptor regulatory network in ovarian cancer
Source: Oncogenesis. 2019 Aug 12;8(8):41. doi: 10.1038/s41389-019-0150-2 (PMC6690894; doi:10.1038/s41389-019-0150-2)
Supplement: Supplementary file 9 — Supplementary Table 3 [file 41389_2019_150_MOESM9_ESM.doc]

Supplementary Table 3: List of antibodies used for western blot and ChIP assays.

| **ASSAY** | **ANTIBODY** | **BRAND** | **CATALOGUE** |
| --- | --- | --- | --- |
| **Western Blot** | Anti-CTCFL | SIGMA Aldrich | HPA001472 |
|  | Anti-GAPDH | Santa Cruz Bt. | SC-25778 |
|  | HRP anti-rabbit | Santa Cruz Bt. | SC-2004 |
| **ChIP** | Anti-BORIS | Santa Cruz Bt. | SC-377085 |
|  | Anti-CTCF | Millipore | 07-729 |
