# Supplementary material for: The epigenetic factor BORIS (CTCFL) controls the androgen receptor regulatory network in ovarian cancer
Source: Oncogenesis. 2019 Aug 12;8(8):41. doi: 10.1038/s41389-019-0150-2 (PMC6690894; doi:10.1038/s41389-019-0150-2)
Supplement: Supplementary file 10 — Supplementary Table 4 [file 41389_2019_150_MOESM10_ESM.doc]

Supplementary Table 4: Sequence of the oligonucleotides used for RT-qPCR and ChIP assays.

| **ASSAY** | **TARGET Gene** | **Sequence (5’-3’)** |
| --- | --- | --- |
| **RT-qPCR** | BORIS | F: GGAGCATTTGTAAACAGTCGGG  R: ATGACCGCTCACATTCGTACC |
|  | BORIS *sf1* | F: GAACGGAGACGAAGCTGCAG  R: CTCTGCAGGCGACAGGAAAC |
|  | *AR* | F: TGCAGCCTATTGCGAGAGAG  TAGATGGGCTTGACTTTCCCAG |
|  | *FN1* | F: TGATGGGAAGACATACCACGTAG  R: TCTGCGGCAGTTGTCACAG |
|  | *FAM129A* | F: GCAAACCAGAGCTTCAGAAATACG  R: GAGTTTCATCAAGCAGGATCTGA |
|  | *CD97* | F: CTCAACAAGAAGCTTCGGGAAG  R: AGAACCATGCGCCTTCATATGC |
|  | *GAPDH* | F: TGTCAAGCTCATTTCCTGGT  R: TCTTACCTCCTTCCAGGCCAT |
| **ChIP** | *AR* | F: TGGCTTGCTCCTCAGTTTGT  R: GCCAGGGGTTCTTTTCAGGA |
|  | *NY-ESO-1* | F: TGGAACTGCATGTCTGGTGG  R: CAGGGACAGAACCCGTTGAA |
